# Pedigree-based QTL analysis of flower size traits in two multi-parental diploid rose populations

**DOI:** 10.3389/fpls.2023.1226713

**Published:** 2023-08-15

**Authors:** Zena Rawandoozi, Ellen L. Young, Shuyin Liang, Xuan Wu, Qiuyi Fu, Tessa Hochhaus, Muqing Yan, Maad Y. Rawandoozi, Patricia E. Klein, David H. Byrne, Oscar Riera-Lizarazu

**Affiliations:** ^1^ Department of Horticultural Sciences, Texas A&M University, College Station, TX, United States; ^2^ Norman Borlaug Institute for International Agriculture and Development, Texas A&M AgriLife Research, Texas A&M System, College Station, TX, United States

**Keywords:** *Rosa*, flower size, FlexQTL, haplotype, pedigree-based analysis

## Abstract

Rose (*Rosa* spp.) is one of the most economically important ornamental species worldwide. Flower diameter, flower weight, and the number of petals and petaloids are key flower-size parameters and attractive targets for DNA-informed breeding. Pedigree-based analysis (PBA) using FlexQTL software was conducted using two sets of multi-parental diploid rose populations. Phenotypic data for flower diameter (Diam), flower weight (fresh (FWT)/dry (DWT)), number of petals (NP), and number of petaloids (PD) were collected over six environments (seasons) at two locations in Texas. The objectives of this study were to 1) identify new and/or validate previously reported QTL(s); 2) identify SNP haplotypes associated with QTL alleles (*Q*-/*q-*) of a trait and their sources; and 3) determine QTL genotypes for important rose breeding parents. Several new and previously reported QTLs for NP and Diam traits were identified. In addition, QTLs associated with flower weight and PD were identified for the first time. Two major QTLs with large effects were mapped for all traits. The first QTL was at the distal end of LG1 (60.44–60.95 Mbp) and was associated with Diam and DWT in the TX2WOB populations. The second QTL was consistently mapped in the middle region on LG3 (30.15–39.34 Mbp) and associated with NP, PD, and flower weight across two multi-parent populations (TX2WOB and TX2WSE). Haplotype results revealed a series of QTL alleles with differing effects at important loci for most traits. This work is distinct from previous studies by conducting co-factor analysis to account for the DOUBLE FLOWER locus while mapping QTL for NP. Sources of high-value (*Q*) alleles were identified, namely, ‘Old Blush’ and *Rosa wichuraiana* from J14-3 for Diam, while ‘Violette’ and PP-J14-3 were sources for other traits. In addition, the source of the low-value (*q*) alleles for Diam was ‘Little Chief’, and *Rosa wichuraiana* through J14-3 was the source for the remaining traits. Hence, our results can potentially inform parental/seedling selections as means to improve ornamental quality in roses and a step towards implementing DNA-informed techniques for use in rose breeding programs.

## Introduction

The rose (*Rosa* spp.) is the queen of flowers and ranks culturally and economically among the most important ornamental plants (Krüssmann, 1981). Roses have been used as garden plants, cut flowers, and for food/medicine/fragrance industrial products for over 5,000 years (Zlesak, 2006). Garden roses accounted for significant ornamental plant sales in the United States (~ $168 million) in 2019 (USDA, 2020).

*Rosa* is an important genus within the Rosaceae family, with a long history of cultivation and breeding, a large area of origin, and an abundance of morphological and adaptation variation. Roses have been bred for many aesthetic traits (e.g., the color, number of petals, floral scent, and prickle formation) for centuries resulting in the diversity seen in our commercial roses today (De Vries and Dubois, 1978; Debener, 2003; Hibrand-Saint Oyant et al., 2008; Gitonga et al., 2014; Schulz et al., 2016; Bourke et al., 2018; Hibrand Saint-Oyant et al., 2018; Zhou et al., 2020; Schulz et al., 2021). Breeding for these traits poses several challenges. In addition to the highly heterozygous nature of the *Rosa* genus (Crespel et al., 2002), the aesthetic attributes are genetically complex, with multiple loci influencing the expression of each trait (Schulz et al., 2021). Moreover, environmental factors play a significant role, necessitating multi-year/season phenotyping efforts (Bourke et al., 2018). Consequently, traditional breeding is time consuming and demands significant effort and resources (Bendahmane et al., 2013; Roman et al., 2015). Therefore, molecular breeding approaches would increase breeding efficiency and accelerate the breeding process (Smulders et al., 2019).

Heat stress is a major constraint for producing crops worldwide, especially in subtropical climates like Texas (Wahid et al., 2007). As for roses, high temperature causes leaf damage, flower abscission, and decreased flower size (flower diameter, petal number) and quality (Moe and Kristoffersen, 1969; Byrne et al., 1978; Zieslin, 1992; Chmelnitsky et al., 2001; Shin et al., 2001; Gitonga et al., 2014; Greyvenstein et al., 2014; Greyvenstein et al., 2015; Liang et al., 2017a; Liang et al., 2017b), significantly reducing market value (Marissen, 2001; Wahid et al., 2007).

The number of petals (NP) is an important ornamental trait in roses, and the more petals a rose has, the more desirable it is for decoration and display purposes. Quantitatively, NP shows low to high narrow sense heritability (0.12–0.88) (Koning-Boucoiran et al., 2012; Liang et al., 2017a; Liang et al., 2017b) and moderately high to high broad sense heritability (0.70–0.96) (Gitonga et al., 2014; Roman et al., 2015; Liang et al., 2017a; Liang et al., 2017b). In wild roses, the majority of genotypes have a single form with five petals (one whorl). In contrast, modern roses frequently have a double flower with various patterns (Reynolds and Tampion, 1983). The increase in petal number is associated with a homeotic conversion in organ identity, in which stamens are transformed into petals (Dubois et al., 2010). The double-flower characteristic in roses is determined by the dominant gene located at the DOUBLE FLOWER locus positioned on chromosome 3 between 33.24 and 33.55 Mb on the *Rosa chinensis* genome v1.0 assembly (Hibrand Saint-Oyant et al., 2018). This gene converts a single flower with one whorl of petals into a double flower with two or more whorls of petals (Debener and Mattiesch, 1999; Rajapakse et al., 2001; Crespel et al., 2002; Linde et al., 2006; Hibrand-Saint Oyant et al., 2008; Spiller et al., 2011; Koning-Boucoiran et al., 2012).

An earlier study suggested that a restricted expression domain of the rose ortholog of AGAMOUS was responsible for the transformation of stamens into petals in the double flowers (Dubois et al., 2010). However, a recent study proposed that misregulation of the rose APETALA2/TOE homolog is responsible for the RhAGAMOUS transcript level reduction, leading to the double flower phenotype (Hibrand Saint-Oyant et al., 2018). This gene has a crucial role in establishing the floral meristem and the specification of floral organs (Bowman et al., 1989; Bowman et al., 1993; Jung et al., 2014) and was mapped in the same region on LG3 (33.23 to 33.24 Mbp) as the major locus of the double flower on the *Rosa chinensis* genome (Hibrand Saint-Oyant et al., 2018).

Beyond this major locus, QTLs for NP have been mapped on all seven LGs using diploid and tetraploid roses. However, the most significant QTL overlapped with the DOUBLE FLOWER locus on chromosome 3 (Linde et al., 2006; Spiller et al., 2011; Koning-Boucoiran et al., 2012; Roman et al., 2015; Hibrand Saint-Oyant et al., 2018; Schulz et al., 2021; Yu et al., 2021).

Flower size traits such as Diam and DWT have a low to moderate narrow sense heritability (*h^2^*) (0.24–0.53) (Liang et al., 2017a; Liang et al., 2017b) and a moderately high to high broad-sense heritability (*H^2^*) (0.62–0.88). A recent study with tetraploid rose reported four QTLs for flower diameter on LGs 2, 4, and 7. The major locus was mapped in a terminal end of LG2 with data for two consecutive years (Yu et al., 2021).

No QTLs associated with either flower weight (fresh/dry) or petaloid number have been reported. A petaloid is an irregular petal shape (also known as petaloid stamens). The phenomenon of petaloid stamens is more common in double flowers and caused by the mutation, downregulation, or expressional boundary shift of AGAMOUS homologs. Petaloid stamens have been reported in several species besides roses (Galimba et al., 2012; Bendahmane et al., 2013; Liu et al., 2013; Noor et al., 2014; Sun et al., 2014; Ma et al., 2015; Mizunoe and Ozaki, 2015; Nakatsuka and Koishi, 2018; Li et al., 2022b). A recent study suggested that the transcription factor RhMYB123 is involved in the development of petaloid stamens in roses (Li et al., 2022a). Moreover, a previous study reported that PD morphology was affected by temperatures indicating that low temperatures induced petaloid stamens in roses (Ma et al., 2015; Li et al., 2022b).

Overall, flower-size traits and petal number are attractive targets for DNA-informed breeding (e.g., marker-assisted parent selection and marker-assisted seedling selection); thus, more studies are needed to elucidate the genetic basis for these traits. In this study, two sets of multi-parental diploid rose populations were used to analyze flower size traits (flower diameter, flower weight, number of petals, and number of petaloids) to 1) detect new and/or validate reported QTLs; 2) identify SNP haplotypes associated with various QTL alleles; and 3) estimate QTL genotypes for rose breeding parents. For this, we performed QTL analysis using pedigree-based analysis (PBA) (Bink et al., 2012; Bink et al., 2014). Our study will increase the understanding of the genetic control and set the stage for molecular breeding of these traits.

## Materials and methods

### Plant material

This study was conducted on two multi-parental diploid rose populations (TX2WOB and TX2WSE). The TX2WOB population was developed from crosses involving breeding lines (J14-3, J3-6, J4-6, and M4-4) derived from *Rosa wichuraiana* ‘Basye’s Thornless’ crossed with ‘Old Blush’, ‘Red Fairy’, ‘Sweet Chariot’, ‘Vineyard Song’, and ‘Little Chief’ (**Supplementary Figure 1**) (Dong et al., 2017; Yan et al., 2019). The TX2WSE population was developed from crosses among *R. wichuraiana* ‘Basye’s Thornless’-derived breeding lines (TAMU7-20, TAMU7-30, J14-3, and M4-4) and ‘Papa Hemeray’, ‘Srdce Europy’, ‘Ole’, *R. setigera*-ARE, and *R. palustris* f. *plena* EB-ARE (**Supplementary Figure 2**) (Young et al., 2022). In addition, these studied diploid populations are segregating for the miniature phenotype (small leaves and flowers).

Five F_1_ TX2WOB rose populations (387 plants) were planted in 2010 at the Horticulture Farm of Texas A&M University in College Station, TX, USA (30.63, −96.37) in one replication and phenotyped in 2015. A subset from TX2WOB (N = 300) was planted in 2018 at the Horticulture Teaching Research and Extension Center (HortTREC) in Somerville, TX (30.524591, −96.422479) with two replications and evaluated in 2021 (**Supplementary Table 1A**). Six F_1_ TX2WSE populations (N = 353) were planted in 2018 at HortTREC with two replications and phenotyped in 2021(**Supplementary Table 1B**). More details on populations and field conditions are described by Rawandoozi et al. (2022) for TX2WOB and Young et al. (2022) for TX2WSE.

### Weather data

Temperature data were obtained from both field locations in 2015 and 2021 (Weather Underground, 2018). The data showed that the temperature varied among seasons and years. The average maximum temperature in the summer season (June, July, and August) in 2015 was hotter than in 2021 (34.1°C vs. 33.3°C). Similarly, 2015 had cooler spring and fall seasons than 2021 (**Supplementary Table 2**; **Supplementary Figure 3**).

### Phenotypic traits

In 2015, the flower size parameters, including flower diameter (Diam), flower dry weight (DWT), and the number of petals (NP), were measured during three seasons [spring (April), summer (August), and fall (November)]. Phenotypic data were collected from at least three fully open flowers randomly chosen in each plant. Flower diameter (cm) was measured in the field, whereas the other two traits were measured in the lab. Flower dry weight (mg) was taken after the whole flower without the pedicel was dried for at least 3 days at 80°C. The number of petals included full-size petals and petaloids (irregularly shaped petals). In 2021, the phenotypic data for flower size were taken from four flowers per plant in the summer (June and July). Diam and NP were measured as described above. Additionally, in this year (2021), both flower fresh weight (mg) (FWT) and the number of petaloids (PD) were measured. The least-square mean (lsmean) of the phenotypic data was used in the statistical analyses and was estimated using “emmeans” v. 1.7.5 package of R (v. 4.1.2; R Foundation for Statistical Computing, Vienna, Austria).

FlexQTL has the functionality to include co-factors and was utilized in the analysis of NP. Since the major QTL for NP was located in the same genomic region harboring the DOUBLE FLOWER locus on LG3, the latter was considered as a covariate in the analysis. Thus, individuals with flowers with fewer than eight petals were considered to have single flowers, whereas those with eight or more petals were considered to have “double” flowers and were given the values of 1 or 2, respectively.

### Heritability and genotype by environment interaction (G×E)

A Shapiro–Wilk test was performed to test the normality of raw and transformed data. Heritability was only estimated for traits measured in 2015 as the data was taken in three seasons using mixed models with a restricted maximum likelihood (REML) estimation method in JMP Pro version 13.2 (SAS Institute Inc., Cary, NC. USA), treating all effects as random (Littell et al., 1996). The following model was used:


$$\begin{array}{c} y=\mu +\sigma_{FP}^{2}+\sigma_{MP}^{2}+\sigma_{Progeny\left(FP,MP\right)}^{2}+\sigma_{Env}^{2}\\ +\sigma_{FP\times Env}^{2}+\sigma_{MP\times Env}^{2}+\sigma_{Progeny\times Env}^{2}+\sigma_{error}^{2} \end{array}$$

where μ is the mean; σ^2^_FP_ and σ^2^_MP_ are the female (FP) and male (MP) parent variances, respectively; σ^2^_Progeny(FP, MP)_ is the progeny variance; σ^2^_Env_ is the environmental variance (seasons); σ^2^_FP × Env_, σ^2^_MP × Env_, and σ^2^_Progeny × Env_ are variances due to the interaction of female and male parents and progenies with the season; and σ^2^_error_ is the error variance.

The sum of the parental variances (σ^2^_FP_ and σ^2^_MP_) was treated as an additive variance ( $\sigma_{A}^{2}$ ), progeny variance [σ^2^_Progeny(FP,MP)_] was as considered nonadditive variance ( $\sigma_{d}^{2}$ ), and the sum of the parental and progeny variances was regarded as the genotypic variance ( $\sigma_{g}^{2}$ ). The interaction of genotype [σ^2^_FP_, σ^2^_MP_, and σ^2^_Progeny(FP,MP)_] by environment (season) was treated as the genetic–environmental variance ( $\sigma_{g\times e}^{2}$ ). The residual variance, confounded with progeny × environmental variance, was regarded as the error variance (σ^2^_error_).

Broad sense heritability for each set of populations across environments was calculated as:


$$\;H^{2}=\frac{\sigma_{g}^{2}\;}{\sigma_{g}^{2}+\frac{\sigma_{g\times e}^{2}}{E}}$$

where *E* indicates the number of environments (seasons) (Holland et al., 2003; Liang et al., 2017b; Wu et al., 2019; Rawandoozi et al., 2021).

The genotype by environment variance to the genetic variance ratio was estimated as:


$$\sigma_{g\times e}^{2}/\sigma_{g}^{2}.$$

A genotype and genotype-by-environment (GGE) biplot analysis was employed to understand the variation due to genotype using the R package “GGEbiplots” v. 0.1.3. Pearson correlation coefficient among phenotypic traits and seasons/years was calculated.

### Genotyping and consensus map development

Two consensus maps were developed for each diploid rose population. The TX2WOB consensus map (415 individuals) was constructed from five rose populations (**Supplementary Table 3**). The TX2WSE was created from three rose populations (314 individuals) (**Supplementary Table 4**).

Genomic DNA was extracted from new rose leaves using Doyle’s CTAB protocol (Doyle and Doyle, 1991). In this study, genotyping by sequencing (GBS) was accomplished using the digital genotyping procedure according to the method described by Morishige et al. (2013). Single-end sequencing was performed using an Illumina HiSeq 2500 platform. The CLC Genomics Workbench v9.0 (Qiagen, Boston, MA) was used to align the reads to the *Rosa chinensis* v1.0 genome (Hibrand Saint-Oyant et al., 2018). Markers were named based on their position in the rose genome and grouped into bins based on their proximity to a given restriction enzyme cut site (NgoMIV) in the reference genome (Yan et al., 2018).

For TX2WOB, before the five individual linkage maps were developed, low-quality SNP markers were eliminated. Tassel version 5 was used to remove markers if they were mapped to chromosome 0, non-biallelic, and markers that had >10% missing data. After that, a Microsoft Excel-based tool and custom R scripts were employed to remove markers with inheritance errors. Then, individual maps were developed for each population using the R package “polymapR” v. 1.1.1, which was set to remove duplicated and distorted markers (p ≥ 0.001). The datasets for each population were simplified by choosing one marker of each marker class per restriction-enzyme bin, which is defined as the region around a NgoMIV cut site, giving preference to markers that are common between populations, having little missing data, and fitting expected segregation ratios. Next, the consensus map was developed using the R package “LPmerge” v. 1.7. The R packages “LinkageMapView” v. 2.1.2 and MapChart software v. 2.32 were utilized to visualize the consensus map. Additional marker curation was performed in FlexQTL software v. 0.1.0.42 to identify and fix/remove problematic markers (singletons, double recombinations, and inheritance errors).

Regarding TX2WSE, the similar procedures described above were used to generate a linkage map, with the exception of filtering markers in PLINK v. 1.9 to eliminate Mendelian-inconsistent mistakes per population. More information about the linkage map development can be found in Rawandoozi et al. (2022) and Young et al. (2022).

### QTL mapping and haplotype analyses

QTL detection via pedigree-based analysis (PBA) was implemented through the FlexQTL software (Bink et al., 2012; Bink et al., 2014). The dataset for the TX2WOB population includes phenotypic data collected from three seasons in 2015 (spring, summer, and fall), the average of three seasons (Mean 2015), and one season in 2021 (summer), along with 1,115 SNP markers. The dataset for the TX2WSE population consists of phenotypic data from one season (summer 2021) and 866 SNP markers.

First, traits were analyzed with a mixed (additive and dominance) model. Then, due to the absence of a dominant effect, QTL analysis was performed in the only additive model at least three times with different parameter settings to ensure reproducibility (Verma et al., 2019). Markov Chain Monte Carlo (MCMC) simulations length ranged from 100,000 to 1,600,000 iterations to store 1,000 samples with thinning between 100 and 1,600.

Convergence was evaluated visually by trace and intensity plots. Twice the natural logarithm of Bayes Factors [2ln(BF)] obtained from FlexQTL software was used to determine the strength of QTLs (Kass and Raftery, 1995). The 2ln(BF) value >2, 5, and 10 indicates positive, strong, and decisive evidence, respectively (Kass and Raftery, 1995; Bink et al., 2014). This study considered major loci if those QTLs were mapped for at least two data sets with 2lnBF ≥ 5, overlapping intervals, and explaining at least 10% of the phenotypic variation. In this study, the physical positions of all mapped QTLs for a given trait(s) were compared across environments and populations. In addition, within each population, if the QTL intervals clustered in the same genomic regions for a trait, these QTLs were considered the same QTL.

The additive ( $\sigma_{A\left(trt\right)}^{2})$ , phenotypic $\left(\sigma_{P}^{2}\right)$ , and residual $\left(\sigma_{e}^{2}\right)$ variances were obtained for each trait from FlexQTL output and used to estimate the narrow-sense heritability (*h^2^*), and the total phenotypic variance explained (PVE) by a QTL was calculated as follows:


$$h^{2}=\frac{\sigma_{A\left(trt\right)}^{2}\;}{\sigma_{P}^{2}}\times 100\;$$

where: $\sigma_{A\left(trt\right)}^{2}$ is the variance of the trait


$$PVE=\frac{\sigma_{A\left(qtl\right)}^{2}\;}{\sigma_{P}^{2}}\times 100\;$$

where: $\sigma_{A\left(qtl\right)}^{2}$ is the variance of QTL

QTLs were named using the Genome Database for Rosaceae QTL naming conventions (Jung et al., 2019). For example, *q*NP.TX2WOB-LG3.1, where *q* stands for QTL, “NP” is the phenotypic trait name (number of petals), “TX2WOB” or “TX2WSE” is the population name that used to develop the consensus map, “LG3” is the linkage group number, and numbers “1” or “2” are used in case there was more than one QTL detected within the same LG. A “CF” suffix was added to the QTL name to distinguish mapping based on co-factor analysis.

SNPs within the region of major QTL(s) (proximity to QTL peaks) for each trait were chosen for haplotype analysis. FlexQTL and the R package “PediHaplotyper” v. 1.0 were used to construct haplotypes (Voorrips et al., 2016). Combinations of diplotypes were used to infer haplotype effects. Diplotype effect differences were evaluated using Steel–Dwass non-parametric multiple comparison test JMP Pro version 13.2. QTL allele groups (*Q* or *q*) were assigned to haplotypes based on their effects. In the case of multi-allelic series, *Q* and *q* alleles were differentiated by an index number.

Haplotypes were traced through pedigrees records. Haplotypes that were traced to a common ancestor were considered identical by descent (IBD), whereas haplotypes that could not be traced to a known common ancestor were defined as identical by state (IBS).

## Results

Phenotypic data for flower size traits were collected from two populations and 2 years. In the TX2WOB population, 387 individuals were measured across three environments (seasons) in 2015, while 277 individuals of TX2WOB and 169 TX2WSE progenies were phenotyped in one environment in 2021 (**Supplementary Tables 1A, B**). For Diam, the data across all data sets were normally distributed (**Supplementary Figure 4**). The highest (4.1 cm) and the lowest (3.3 cm) mean Diam were recorded in fall 2015 (TX2WOB) and summer 2021 (TX2WSE), respectively (**Supplementary Table 5**). The flower weight (dry and fresh weight) data were not normally distributed, except for the mean 2015 for DWT (**Supplementary Figure 5**). In 2015, the highest DWT was in fall (9.0 mg), and the lowest was in summer (6.4 mg). In 2021, the highest mean FWT (280 mg) was observed in TX2WOB, and the lowest (190 mg) was in TX2WSE (**Supplementary Table 5**).

All NP data sets were skewed towards low NP (less than eight petals, single flower) (**Supplementary Figure 6A**). The highest mean NP (27.3) was in the fall of 2015, whereas the lowest (14.1) was in the summer of 2021 for TX2WOB and TX2WSE, respectively (**Supplementary Table 5**). Generally, across all individual populations in TX2WOB and TX2WSE and regardless of the season, most petal data sets showed a bimodal distribution with either single or double flower group, and a few of them showed a trimodal distribution with peaks corresponding to single-flower (less than eight petals), semi- (8–40 petals), and double-flower (over 40 petals) categories (Dubois et al., 2010) (data not shown). In addition, the segregation ratio of progenies from each population comprising TX2WOB and TX2WSE indicates that a single dominant locus controls single to double petals is present in these populations.

Lastly, the two PD data sets were skewed towards low PD in both populations (**Supplementary Figure 6B**). The mean of PD for the TX2WOB population was 2.6, while the mean for the TX2WSE population was 1.9 (**Supplementary Table 5**). In addition, this study unveiled moderate to moderately high correlations (r= 0.40 and 0.75) between PD and the double flowers type in both populations (data not shown).

Overall, warmer temperatures (~27–29°C) during the summer months of 2015 and 2021 were associated with smaller flower sizes and fewer petals than the cooler temperatures observed during the spring and fall months (**Supplemental Table 2**; **Supplementary Figure 3**).

### Heritability and G×E interaction

The narrow sense heritability (*h* ^2^) varied among traits, environments, and populations. It was low for PD (0.16–0.39); low to moderate for Diam (0.31–0.56), FWT (0.38–0.46), and DWT (0.40–0.57); and low to moderately high for NP (0.38–0.74) (**Table 1**). The broad sense heritability was moderately high to high (0.75–0.87) for Diam, DWT, and NP (**Supplementary Table 6**).

**Table 1:** QTLs mapped for the diameter (Diam), dry weight (DWT), fresh weight (FWT), number of petals (NP), and petaloids (PD) phenotyped in Texas on five diploid rose populations (TX2WOB) across multiple seasons in 2015 in College Station and on 10 populations of TX2WOB and six populations of TX2WSE in summer 2021 in Somerville.

Trait	Population	Season	μ	σ^2^_p_	σ^2^_e_	σ^2^_A_	h^2^	LG	BF		
									1/0	2/1	3/2
Diam	TX2WOB	Spring 2015	3.8	0.39	0.21	0.18	0.47	1	13.4	0.0	−4.2
								2	8.1	−0.5	−2.5
	TX2WOB	Summer 2015	3.6	0.41	0.18	0.23	0.56	2	6.5	1.2	−0.1
								3	2.0	0.1	NA
								5	7.3	1.8	−0.8
								6	2.6	−0.9	NA
	TX2WOB	Fall 2015	4.1	0.58	0.30	0.28	0.48	1	29.2	1.0	−0.6
								2	8.6	0.7	−3.4
								3	2.8	−1.5	NA
	TX2WOB	Mean 2015	3.7	0.46	0.25	0.21	0.45	1	10.7	0.2	−0.2
								2	5.4	2.2	−1.6
								6	3.2	−1.0	NA
	TX2WOB	Summer 2021	3.5	0.31	0.21	0.10	0.31	2	5.4	0.8	−1.6
								4	3.4	−0.2	−0.9
								6	5.0	0.0	−1.7
	TX2WSE	Summer 2021	3.3	0.32	0.19	0.13	0.41	2	6.2	0.9	0.1
								3	4.3	1.3	−0.9
								7	9.8	1.8	0.4
DWT	TX2WOB	Spring 2015	7.2	7.92	4.79	3.13	0.40	1	6.7	−1.5	NA
								3	27.9	3.3	0.5
	TX2WOB	Summer 2015	6.4	10.21	4.43	5.78	0.57	3	29.2	−0.3	−1.8
								5	4.2	0.6	−0.7
	TX2WOB	Fall 2015	9.0	15.39	8.41	6.98	0.45	1	6.3	0.9	−1.9
								3	15.1	1.1	−1.9
	TX2WOB	Mean 2015	7.5	10.91	5.20	5.71	0.52	1	29.7	−2.3	NA
								2	3.8	−3.5	−0.6
								3	NA	30.7	−0.6
FWT	TX2WOB	Summer 2021	300.0	30.00	20.00	10.00	0.38	3	26.6	5.1	1.5
	TX2WSE	Summer 2021	200.0	10.00	10.00	10.00	0.46	3	26.2	2.4	2.7
								5	3.1	−0.3	−0.5
NP	TX2WOB	Spring 2015	20.3	232.27	66.82	165.45	0.71	3	28.8	1.4	0.3
	TX2WOB	Summer 2015	23.8	355.43	151.28	204.15	0.57	3	26.0	5.5	2.7
	TX2WOB	Fall 2015	27.3	825.27	256.01	269.27	0.69	3	25.6	5.5	3.3
	TX2WOB	Mean 2015	23.5	382.50	100.00	282.50	0.74	2	7.1	−1.8	NA
								3	NA	13.3	3.2
	TX2WOB	Summer 2021	22.4	278.65	171.51	107.14	0.38	3	28.4	2.2	1.3
	TX2WSE	Summer 2021	14.1	141.80	41.34	100.47	0.71	3	NA	29.6	−0.4
NP (co-factor)	TX2WOB	Spring 2015	20.3	232.27	57.51	174.76	0.75	3	28.8	1.3	0.4
	TX2WOB	Summer 2015	23.8	355.43	114.59	240.84	0.68	3	10.6	2.4	0.8
	TX2WOB	Fall 2015	27.3	825.27	202.33	622.95	0.75	3	9.4	3.6	1.9
	TX2WOB	Mean 2015	23.5	382.50	127.02	255.48	0.67	2	2.8	−0.1	−2.5
								3	28.4	2.4	0.0
	TX2WOB	Summer 2021	22.4	278.65	135.15	143.50	0.51	3	14.9	1.5	0.8
	TX2WSE	Summer 2021	14.1	141.80	32.66	109.15	0.77	3	28.3	−2.3	NA
PD	TX2WOB	Summer 2021	2.6	5.01	4.20	0.81	0.16	3	28.7	1.7	0.5
	TX2WSE	Summer 2021	1.9	4.40	2.68	1.73	0.39	3	27.8	0.4	−0.4

Markov chain Monte Carlo (MCMC) run length, phenotypic mean (μ), phenotypic variance (σ^2^_P_), residual variance(σ^2^_e_), additive variance(σ^2^_A_), narrow-sense heritability (h^2^), and the linkage groups (LG) that QTLs were mapped on.

2ln(BF). Bayes factor quantifies the support from the data for the number of QTL(s) in the model (QTL evidence), after pair-wise model comparison (1/0, 2/1, and 3/2) such as “one-QTL model” vs. “zero-QTL model. 2ln(BF)<0 = no evidence; 0–2 = hardly any; 2–5 = positive; 5–10 = strong; >10 = decisive. Bayes factor not available (na) if either model does not have enough samples in the Markov chain.

Diam exhibited moderately high broad sense heritability (*H* ^2^ = 0.75), moderate to strong correlations among environments (r = 0.52–0.87) (**Supplementary Table 7**), and a moderate ratio of G×E to G variance ( $\sigma_{\text{g}\times \text{e}}^{2}/\sigma_{\text{g}}^{2}=1.00$ ) (**Supplementary Table 6**). These results suggest the existence of genotype sensitivity to the environment. However, the other two traits, DWT and NP, showed high to very high broad sense heritability (*H* ^2^ = 0.82 and 0.87, respectively), strong correlations among environments (r = 0.70–0.93), and minimal genotype by environment interaction ( $\sigma_{\text{g}\times \text{e}}^{2}/\sigma_{\text{g}}^{2}=0.65$ and 0.43, respectively). These results were further supported by the greater amount of variation explained by the first principal component (PC1) (82.61% and 92.73%) for DWT and NP as compared to Diam (80.83%) (**Supplementary Figure 7**). Furthermore, the GGE biplots for Diam showed that the summer vector was far from the other two seasons, indicating that the high temperatures in the warm season (average monthly summer temperature ~29°C) discriminate genotypes differently than the cool seasons (average monthly spring and fall temperature ~20–22°C). In addition, cool seasons for this trait discriminated genotypes similarly, supported by the short distance between them. This finding is confirmed by the correlations among these three seasons (**Supplementary Table 7**). In contrast, the GGE biplots showed for NP and DWT that fall is more discriminating and discriminates differently than spring and summer seasons.

In general, for both NP and DWT, the $\sigma_{\text{g}\times \text{e}}^{2}/\sigma_{\text{g}}^{2}$ ratio were<1, suggesting that we should be able to make progress in selecting individuals across different seasons as the genetic variance is greater than the G×E variance. In comparison, the selection for Diam is season dependent. Consequently, the selection for Diam during both cool and warm seasons would be preferred to develop a stable flower diameter.

Among traits, the correlation results showed that flower weight, whether it was DWT (TX2WOB, 2015) or FWT (both populations in 2021), was positively correlated with Diam (r=0.27–0.47) and NP (r= 0.53–0.85) (**Supplementary Table 8**). This finding was expected as the flower weight reflected the combination of Diam and NP. A weak and negative correlation was found between Diam and NP, reflecting a weak tendency of the larger flowers to have fewer petals in the studied rose populations.

In addition, high positive correlations were found between FWT and NP with PD in TX2WOB (r= 0.78 and 0.79) and TX2WSE (r= 0.89 and 0.69). Lastly, there were strong positive correlations between the 2 years’ data (2015 and 2021) in TX2WOB for Diam (r=0.66), fresh and dry flower weight traits (r=0.76), and NP (r=0.93) (**Supplementary Table 8**).

### QTL mapping

Several QTLs associated with flower size traits were identified across the two diploid rose populations and six phenotypic data sets (spring, summer, fall, and the mean of 2015 for TX2WOB and summer 2021 for both TX2WOB and TX2WSE), which were evaluated over two locations in Texas (**Table 1**).

### Flower diameter

**Figure 1:**
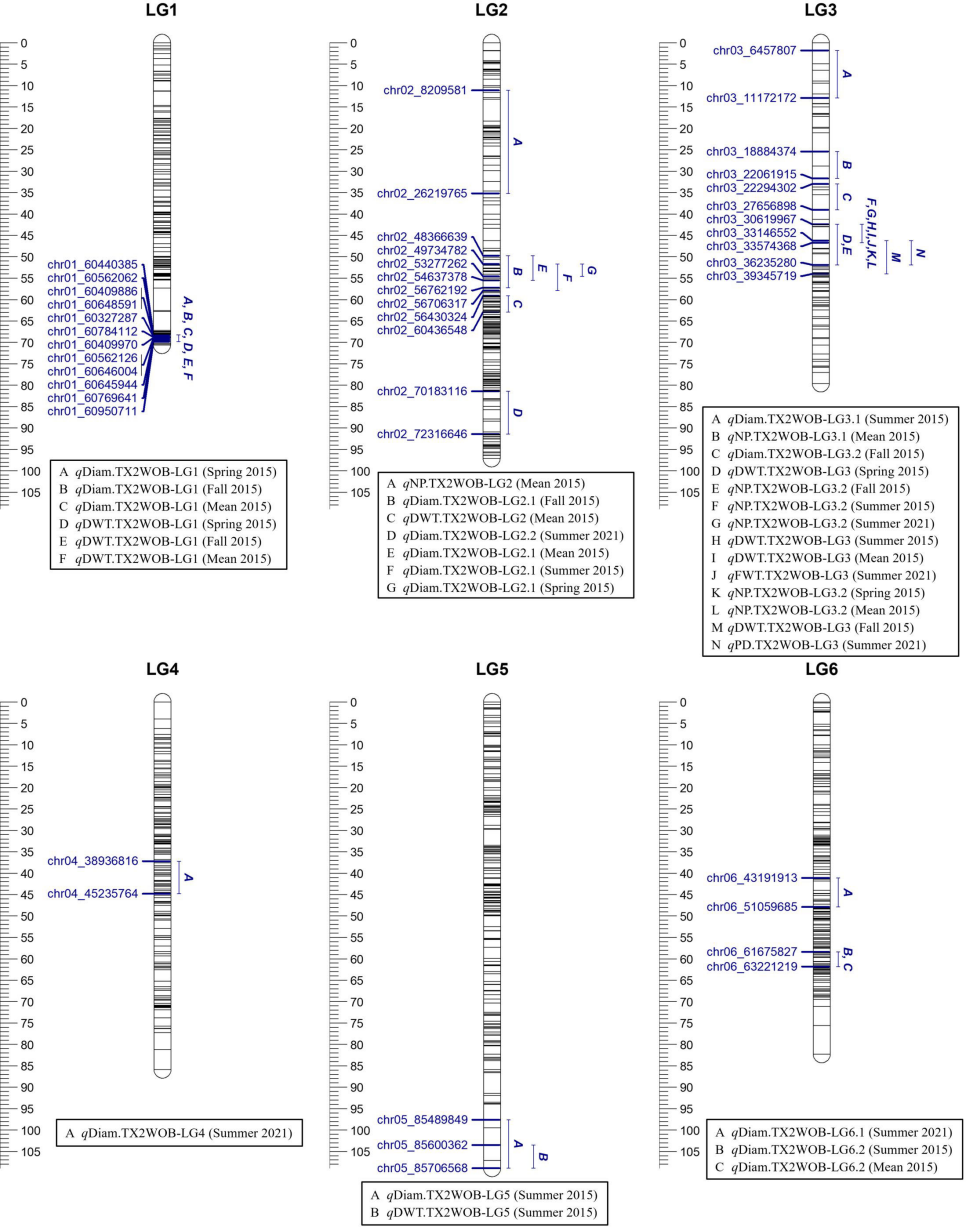
Positions of putative QTLs controlling the diameter (Diam), dry weight (DWT), fresh weight (FWT), number of petals (NP), and number of petaloids (PD) in five diploid rose populations 2015 and on 10 populations in summer 2021 at linkage groups (LG) of the five-population (TX2WOB) consensus map. QTL names are listed below each LG. The plot generated using MapChart 2.32.

For TX2WOB, FlexQTL software detected nine QTLs associated with Diam on all linkage groups (LGs), except LG7, across five seasons (**Table 1**; **Figure 1** and **Supplementary Figure 8**). A major QTL (*q*Diam.TX2WOB-LG1) was consistently detected on the distal end of LG1 across two cool seasons (spring and fall) and mean 2015 data sets between 68.2 and 69.8 cM [60.44–60.95 Mbp on the *Rosa chinensis* genome v1.0 (Hibrand Saint-Oyant et al., 2018)] with mode (peak) at 69.0 cM (**Table 2**; **Figure 1** and **Supplementary Figure 8**). This QTL showed decisive evidence, high posterior intensity, and the phenotypic variation explained (PVE) by this QTL ranged from 67% to 80% (**Tables 1**, **2**). In this study, *q*Diam.TX2WOB-LG1 passed our inclusion criteria.

**Table 2:** QTL name, linkage group (LG), interval, QTL position of the nearest SNP marker to mode (peak), posterior intensity (QTL intensity), and phenotypic variance explained (PVE) for the diameter (Diam), dry weight (DWT), fresh weight (FWT), and number of petaloids (PD) phenotyped in Texas on five diploid rose populations of TX2WOB across multiple seasons in 2015 in College Station (CS) and on 10 population of TX2WOB and six populations of TX2WSE in summer 2021 in Somerville (SV).

QTL name	Season/year	LG	Peak	Interval		QTL intensity	PVE
			cM (Mbp)	(cM)	(Mbp)		(%)
*q*Diam.TX2WOB-LG1	Spring 2015	1	69.0 (60.56)	[68.2–69.8]	[60.44–60.95]	1.03	78
	Fall. 2015	1	69.0 (60.56)	[68.2–69.8]	[60.44–60.95]	1.00	67
	Mean 2015	1	69.0 (60.56)	[68.2–69.8]	[60.44–60.95]	1.07	80
*q*Diam.TX2WOB-LG2.1	Spring 2015	2	53.3 (51.82)	[51.7–54.6]	[49.73–53.27]	0.93	13
	Summer 2015	2	53.3 (51.82)	[51.7–57.9]	[49.73–56.7]	1.08	15
	Fall. 2015	2	55.0 (54.63)	[49.7–57.2]	[48.36–56.76]	0.91	11
	Mean 2015	2	52.0 (51.02)	[49.7–55.5]	[48.36–54.63]	1.00	11
*q*Diam.TX2WOB-LG2.2	Summer 2021	2	85.0 (70.88)	[81.4–91.4]	[70.18–72.31]	0.91	16
*q*Diam.TX2WSE-LG2	Summer 2021	2	114.97 (72.31)	[106.52–114.97]	[71.30–72.31]	1.08	12
*q*Diam.TX2WOB-LG3.1	Summer 2015	3	6.4 (9.46)	[1.8–12.9]	[6.45–11.17]	0.49	14
*q*Diam.TX2WOB-LG3.2	Fall. 2015	3	35.5 (23.49)	[33.0–39.0]	[22.29–27.65]	0.40	9
*q*Diam.TX2WSE-LG3	Summer 2021	3	16.31 (21.51)	[0.00–17.76]	[15.44–23.44]	0.96	12
*q*Diam.TX2WOB-LG4	Summer 2021	4	43.7 (44.88)	[37.2–44.8]	[38.93–45.23]	0.73	12
*q*Diam.TX2WOB-LG5	Summer 2015	5	107.1 (85.62)	[97.6–108.9]	[85.48–85.70]	0.88	18
*q*Diam.TX2WOB-LG6.1	Summer 2021	6	45.2 (49.99)	[41.1–47.9]	[43.19–51.05]	0.83	18
*q*Diam.TX2WOB-LG6.2	Summer 2015	6	60.6 (62.42)	[58.4–61.8]	[61.67–63.22]	0.57	12
	Mean 2015	6	61.1 (63.22)	[58.4–61.8]	[61.67–63.22]	0.40	9
*q*Diam.TX2WSE-LG7	Summer 2021	7	17.80 (1.20)	[13.66–18.78]	[0.40–2.49]	0.53	21
*q*DWT.TX2WOB-LG1	Spring 2015	1	69.0 (60.56)	[68.2–69.8]	[60.44–60.95]	0.93	41
*q*DWT.TX2WOB-LG1	Fall 2015	1	69.0 (60.56)	[68.2–69.8]	[60.44–60.95]	0.83	41
*q*DWT.TX2WOB-LG1	Mean 2015	1	69.0 (60.56)	[68.2–69.8]	[60.44–60.95]	1.00	40
*q*DWT.TX2WOB-LG2	Mean 2015	2	61.1 (58.49)	[59.1–62.9]	[56.43–60.43]	0.53	6
*q*DWT.TX2WOB-LG3	Summer2015	3	46.7 (33.57)	[42.4–46.7]	[30.61–33.57]	1.06	36
	Spring 2015	3	51.9 (36.24)	[46.2–54.0]	[33.14–39.34]	1.16	29
	Fall 2015	3	51.9 (36.23)	[46.2–51.9]	[33.14–36.23]	1.03	24
	Mean 2015	3	43.0 (31.00)	[42.4–46.7]	[30.61–33.57]	1.40	38
*q*DWT.TX2WOB-LG5	Summer 2015	5	108.9 (85.70)	[103.5–108.9]	[85.60–85.70]	0.91	25
*q*FWT.TX2WOB-LG3	Summer 2021	3	43.0 (31.00)	[42.4–46.7]	[30.61–33.57]	1.18	23
*q*FWT.TX2WSE-LG3	Summer 2021	3	25.38 (30.15)	[25.38–33.53]	[30.15–33.83]	0.94	46
*q*FWT.TX2WSE-LG5	Summer 2021	5	42.19 (7.30)	[40.55–47.84]	[7.99–10.26]	0.53	8
*q*PD.TX2WOB-LG3	Summer 2021	3	49.1 (34.21)	[46.2–51.9]	[33.14–36.23]	1.15	17
*q*PD.TX2WSE-LG3	Summer 2021	3	31.88 (34.36)	[29.24–31.88]	[31.09–34.36]	1.20	42

Two minor QTLs were detected on LG2 and LG6. *q*Diam.TX2WOB-LG2.1 was mapped at the middle part of LG2 between 49.7 and 57.9 cM (48.36–56.70 Mbp) in four data sets with strong evidence, and PVE ranged from 11% to 15% (**Tables 1**, **2**; **Figure 1**). *q*Diam.TX2WOB-LG6.2 was detected between 58.4 and 61.8 cM (61.67–63.22 Mbp) in two data sets with positive evidence and PVE of 9%–12%. Most other QTLs identified in this population were environment specific.

**Figure 2:**
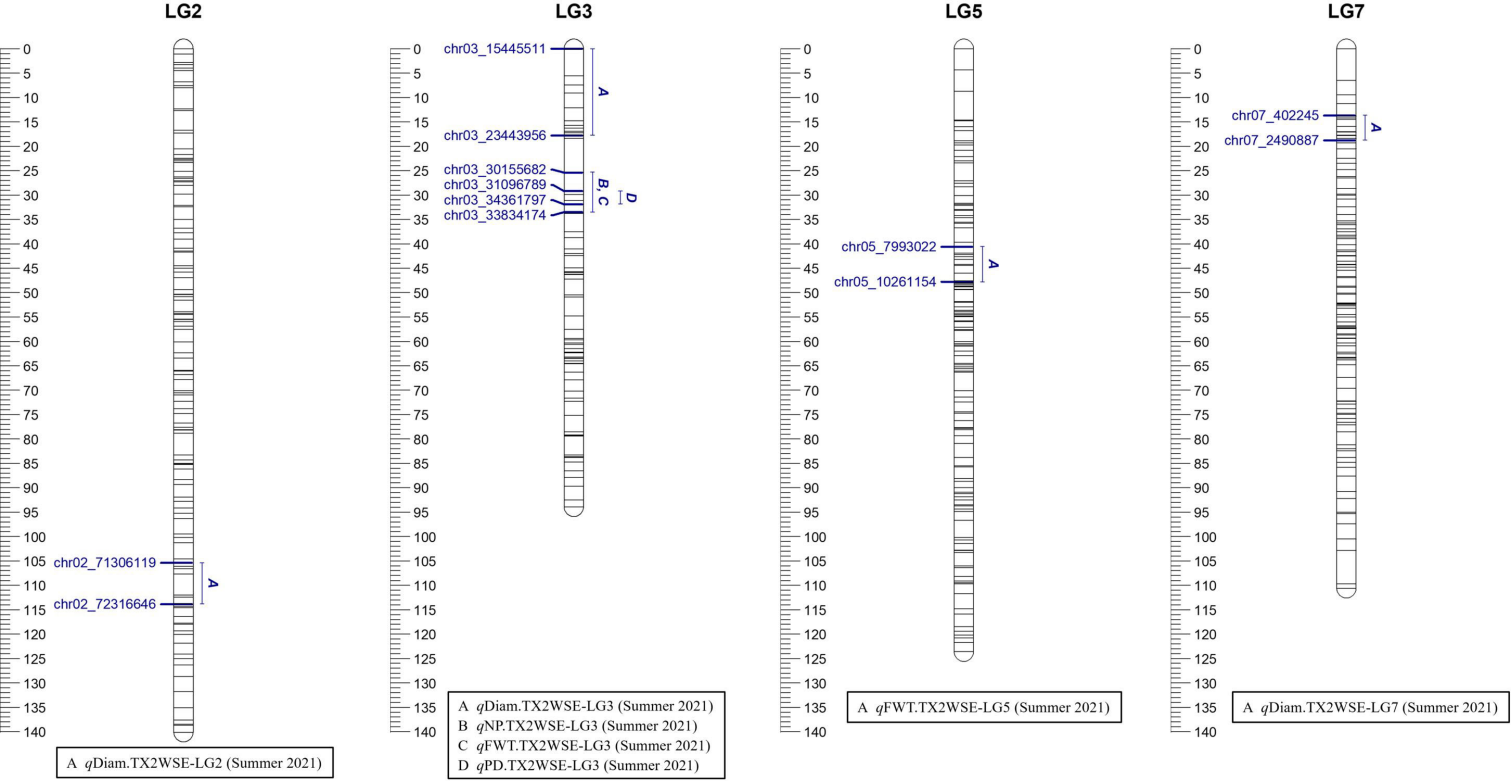
Positions of putative QTLs controlling the diameter (Diam), fresh weight (FWT), number of petals (NP), and number of petaloids (PD) in six diploid rose populations in summer 2021 at linkage groups (LG) of the six-population (TX2WSE) consensus map. QTL names are listed below each LG. The plot generated using MapChart 2.32.

Three QTLs were mapped in the TX2WSE population for Diam with positive/strong evidence on LG 2, 3, and 7 using one data set (summer 2021) (**Table 1**; **Figure 2** and **Supplementary Figure 8**). *q*Diam.TX2WSE-LG2 was between 106.52 and 114.97 cM (71.30–72.31 Mbp) and PVE of 12%. *q*Diam.TX2WSE-LG3 and *q*Diam.TX2WSE-LG7 were at the proximal ends of LG3 and LG7, respectively, with PVE of 12%–21% (**Table 2**).

### Flower weight (dry/fresh)

For the TX2WOB population, four QTLs associated with DWT were identified (**Table 1**; **Figure 1** and **Supplementary Figure 9**). Of these, major QTLs on LG1 and LG3 were consistently detected with either strong or decisive evidence at the same positions in at least two seasons and showed high PVE (**Tables 1**, **2**). Hence, these two QTLs were considered for further analysis. *q*DWT.TX2WOB-LG1 was consistently mapped across three data sets in 2015 at the distal end of LG1 between 68.2 and 69.8 cM (60.44–60.95 Mbp) and mode at 69.0 cM with large effects (PVE, 40%–41%) (**Table 2**; **Figure 1**).

The second major QTL on LG3, *q*DWT.TX2WOB-LG3, was identified at the middle part of LG3 with intervals ranging from 42.4 to 54.0 cM (30.61–39.34 Mbp) across three seasons and the mean in 2015. Peaks for this QTL co-localized at positions between 43.0 and 51.9 cM, with PVE ranging from 24% to 38% (**Table 2**; **Figure 1**). The other two QTLs on LG2 and LG5 were mapped once and considered environment specific.

One major QTL for FWT was mapped with decisive evidence using TX2WOB and one season (summer 2021) on LG3, *q*FWT.TX2WOB-LG3 (**Table 1**; **Figure 1**). The QTL interval ranged from 42.4 to 46.7 cM (30.61–33.57 Mbp) and PVE of 23% (**Table 2**).

On the other population, TX2WSE, two QTLs were mapped for FWT on LG3 and LG5 using one data set (summer 2021) (**Tables 1**, **2**; **Figure 2**). *q*FWT.TX2WSE-LG3 was a major QTL mapped with an interval ranging from 25.38 to 33.53 cM (30.15–33.83 Mbp), decisive evidence, and PVE of 46%.

### Number of petals

For TX2WOB, one to three QTLs for NP were detected per environment on LG3, two of these passed our inclusion criteria (**Table 1**; **Figure 1** and **Supplementary Figure 10**). The first major LG3 QTL that was mapped at the middle part (*q*NP.TX2WOB-LG3.2) was common to all five data sets examined across 2 years, with PVE of 34%–76% (**Tables 1**, **3**; **Figure 1**). Peaks for this QTL co-localized across all environments, having their mode at 43.0, 46.2, and 46.7 cM, and their interval between 42.4 and 46.7 cM (30.61–33.57 Mbp), except for fall 2015, which had wider intervals spanning 42.4–51.9 cM (30.61–36.23 Mbp) (**Table 3**). The second major QTL on LG3, *q*NP.TX2WOB-LG3.1, was located upstream *q*NP.TX2WOB-LG3.2, at 25.4–31.7 cM (18.88–22.06 Mbp) and identified in the fall and the mean 2015 data sets with PVE up to 37% (**Table 3**; **Figure 1**). The LG2 QTL was mapped at the upper part of the LG and was environment specific in this study, as it appeared only once in the dataset.

**Table 3:** QTL name, linkage group (LG), interval, QTL position of the nearest SNP marker to mode (peak), posterior intensity (QTL intensity), and phenotypic variance explained (PVE) for the number of petals (NP) phenotyped in Texas on five diploid rose populations (TX2WOB) across multiple seasons in 2015 in College Station (CS) and on 10 populations of TX2WOB and six populations of TX2WSE in summer 2021 in Somerville (SV).

QTL name	Season/year	LG	Peak	Interval		QTL intensity	PVE
			cM (Mbp)	(cM)	(Mbp)		(%)
Without co-factor							
*q*NP.TX2WOB-LG2	Mean 2015	2	23.4 (11.27)	[11.1–35.2]	[8.20–26.21]	0.85	6
*q*NP.TX2WOB-LG3.1	Fall 2015	3	28.8 (21.40)	[25.4–31.7]	[18.88–22.06]	0.66	36
	Mean 2015	3	28.8 (21.40)	[25.4–31.7]	[18.88–22.06]	1.00	37
*q*NP.TX2WOB-LG3.2	Mean 2015	3	46.2 (33.14)	[42.4–46.7]	[30.61–33.57]	1.02	76
	Fall 2015	3	46.7 (33.57)	[42.4–51.9]	[30.61–36.23]	0.96	44
	Spring 2015	3	46.2 (33.14)	[42.4–46.7]	[30.61–33.57]	1.27	42
	Summer 2015	3	46.2 33.14)	[42.4–46.7]	[30.61–33.57]	1.29	38
	Summer 2021	3	43.0 (31.00)	[42.4–46.7]	[30.61–33.57]	1.30	34
*q*NP.TX2WSE-LG3	Summer 2021	3	31.88 (33.37)	[25.38–33.53]	[30.15–33.83]	1.60	58
With co-factor							
*q*NP.TX2WOB-LG2.CF	Mean 2015	2	67.1 (63.66)	[55.0–67.9]	[53.32–64.33]	0.24	9
*q*NP.TX2WOB-LG3.CF	Mean 2015	3	46.7 (33.57)	[39.0–46.7]	[27.65–33.57]	1.00	20
	Fall 2015	3	28.8 (21.40)	[25.4–51.9]	[18.88–36.23]	0.70	27
	Spring 2015	3	46.7 (33.57)	[39.0–46.7]	[27.65–33.57]	1.12	20
	Summer 2015	3	28.8 (21.40)	[25.4–41.3]	[18.88–29.38]	0.86	25
	Summer 2021	3	46.7 (33.57)	[39.0–46.7]	[27.65–33.57]	0.88	12
*q*NP.TX2WSE-LG3.CF	Summer 2021	3	25.38 (30.15)	[18.39–29.88]	[27.80–29.08]	0.83	41

NP was analyzed with the original data (without co-factor) (top) and using DOUBLE FLOWER locus as a co-factor (bottom).

As for TX2WSE, one major QTL (*q*NP.TX2WSE-LG3) with decisive evidence and large effect (58% PVE) was identified at the middle part of LG3 between 25.38 and 33.53 cM (30.15–33.83 Mbp) with a peak at 31.88 cM (**Tables 1**, **3**; **Figure 2** and **Supplementary Figure 10**).

Moreover, the posterior intensity of *q*NP.TX2WOB-LG3.2 and *q*NP.TX2WSE-LG3 was generally >1 (**Table 3**), which implies that FlexQTL assigned two QTLs within the same QTL interval. This was supported by Bayes factors values, which showed evidence of more than one QTL (**Table 1**). However, the downstream analysis of “iqtl.out” file from FlexQTL output showed that the distance between these QTLs was very short (ranging from 1.7 to 2.9 cM) (data not shown). FlexQTL is not equipped to distinguish QTLs within this short distance.

Since the major QTLs of NP (*q*NP.TX2WOB-LG3.2 and *q*NP.TX2WSE-LG3) colocalized with the DOUBLE FLOWER locus on LG3 in all data sets in both populations, this trait was considered for co-factor analysis to assess the effect of this locus of masking the detection of additional QTLs with minor effects. The results from the co-factor analysis were similar to those of the original data set in TX2WOB. However, the major QTL on LG3 (*q*NP.TX2WOB-LG3.CF) had a larger interval between 39.0 and 46.7 cM (27.65–33.57 Mbp) in most data sets, 25.4 and 51.9 cM (18.88–36.23 Mbp) in fall, and 25.4 and 41.3 cM (18.88–29.38 Mbp) in summer 2015 with PVE of 12%–28% (**Tables 1**, **3**; **Supplementary Figure 11**). In addition, the minor QTL on LG2 was mapped in the middle part of the LG. However, for TX2WSE, a new QTL was mapped upstream to *q*NP.TX2WSE-LG3 between 18.39 and 29.88 cM (27.80–29.08 Mbp) on LG3 (*q*NP.TX2WSE-LG3.CF) with PVE of 41% (**Table 3**). The QTL mapping results using the DOUBLE FLOWER locus as a covariate helped identify an additional major QTL on LG3 for TX2WSE and another minor QTL on LG2 for TX2WOB. In addition, a new chromosomal region was discovered on LG3 for TX2WOB that was confounded with one or both major QTLs (*q*NP.TX2WOB-LG3.1 and *q*NP.TX2WOB-LG3.2).

Regarding PD, one QTL on LG3 with decisive evidence and large effect was mapped for each population using one data set (summer 2021) and was considered for the downstream analysis (**Tables 1**, **2**; **Figures 1**, **2** and **Supplementary Figure 12**). *q*PD.TX2WOB-LG3 was detected between 46.2 and 51.9 cM (33.14–36.23 Mbp) with PVE of 17% in TX2WOB (**Table 2**), whereas *q*PD.TX2WSE-LG3 was between 29.24 and 31.88 cM (31.09–34.36 Mbp) and PVE of 42% (**Table 2**).

For Diam, only two QTL genotypes (*QQ* and *Qq*) were predicted at the peaks of *q*Diam.TX2WOB-LG1 and *q*Diam.TX2WOB-LG2.2, where *q* and *Q* were associated with low and high Diam, respectively (**Supplementary Figures 13A, B**). Progenies were categorized into *QQ* and *Qq* groups with a mean Diam of 3.4 and 2.8 cm for *q*Diam.TX2WOB-LG1, and 3.5 and 3.4 cm for *q*Diam.TX2WOB-LG2.2, respectively. In contrast, the three genotype classes for *q*Diam.TX2WSE-LG2 had Diam means of 3.6, 3.3, and 3.1 cm for *QQ*, *Qq*, and *qq* classes, respectively (**Supplementary Figure 13C**). Generally, progenies with the favorable allele (*Q*) were associated with increasing Diam and were more prevalent in TX2WOB dataset compared to TX2WSE population.

Regarding the flower dry weight for TX2WOB, two QTL genotypes were predicted for both major QTLs. QTL genotypes had an average DWT of 6.4 and 3.2 mg for *q*DTW.TX2WOB-LG1 and 8.5 and 7.2 mg for *q*DTW.TX2WOB-LG3, for progenies having the *QQ* and *Qq* genotypes, respectively (**Supplementary Figures 14A, B**). The two QTL genotypes classes at *q*FTW.TX2WSE-LG3 have estimated mean FWT of 280.7 and 141.3 mg for *Qq* and *qq* genotype classes, respectively (**Supplementary Figure 14C**). No individuals were in the *qq* genotype class in TX2WOB (**Supplementary Figures 14A, B**). Therefore, more favorable alleles (*Q*) are associated with increasing flower weight in TX2WOB as compared to TX2WSE.

Two QTL genotype groups were identified at the LG3 QTLs for NP across the two populations. For TX2WOB, QTL genotypes had an average NP of 26.7 and 11.4 for *q*NP.TX2WOB-LG3.1 and 35.5 and 15.4 for *q*NP.TX2WOB-LG3.2 for progenies having the *Qq* and *qq* genotypes, respectively (**Supplementary Figures 15A, B**). In contrast, progenies were categorized into *Qq* and *qq* groups with a mean NP of 25.8 and 7.4 for *q*NP.TX2WSE-LG3 (**Supplementary Figure 15C**). In summary, unfavorable alleles (*q*) associated with decreasing petals were common in TX2WSE (approximately 50% of progenies). The opposite was true in TX2WOB.

As for PD, three QTL genotypes classes were estimated at *q*PD.TX2WOB-LG3 with a mean of PD 2.9, 2.5, and 2.0 for *QQ*, *Qq*, and *qq* classes, respectively (**Supplementary Figure 16A**). In contrast, individuals were classified into *Qq* and *qq* groups with an average of 3.0 and 0.6 for *q*PD.TX2WSE-LG3, in which half of the individuals had had *q-*allele associated with fewer PD (**Supplementary Figure 16B**).

Thus, lacking or unbalanced representation of QTL genotype classes in most studied traits due to the small sample size of some populations hindered our ability to make robust conclusions about QTL gene action.

### Haplotype analysis for major QTLs

#### Flower diameter

**Figure 3:**
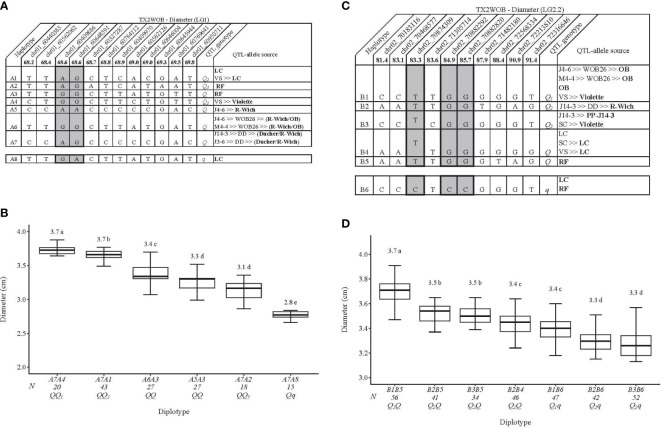
QTL genotypes for diameter in diploid rose breeding parents with haplotype names, SNP sequences, origin sources, and alleles for predictive SNP markers associated with *Q-* or *q*-alleles for increasing or decreasing the diameter, respectively, are shaded **(A, C)**, and the diplotype effect of the most common haplotypes associated with the diameter **(B, D)** at *q*Diam.TX2WOB-LG1 and *q*Diam.TX2WOB-LG2.2, respectively. The *Q* without a subscript indicates that were not able to categorize this haplotype due to the lack of appropriate diplotype combinations. Means not connected by the same letter are significantly different (*p*<0.05) within each population using the non-parametric multiple comparison Steel–Dwass test. N = Diplotype sample size. LC, ‘Little Chief’; VS, ‘Vineyard Song’; RF, ‘Red Fairy’; OB, ‘Old Blush’; SC, ‘Sweet Chariot’; R-Wich, *Rosa wichuraiana*.

For TX2WOB, 12 SNPs in the *q*Diam.TX2WOB-LG1 region (68.2–69.8 cM) spanning 0.5 Mbp (**Figure 3A**) were chosen for haplotyping. Eight SNP haplotypes (A1–A8) across the seven parents in which A8 was the only haplotype associated with decreasing Diam and was assigned to the *q-*allele, while the remaining haplotypes related to increased Diam (*Q-*allele) (**Figure 3A**). The estimation of diplotype effects indicated that A4 (*Q-*allele) had a greater effect in increasing Diam than A1 (*Q-*allele), A2 (*Q-*allele), and A8 (*q-*allele) when comparing the diplotypes of A7A4, A7A1, A7A2, and A7A8 (**Figure 3B**). Thus, multiple *Q-*alleles of different effects exist at this locus. The haplotype effects order was A4>A1>A2 corresponding to *Q_1_*, *Q_2_*, and *Q_3_*, respectively. Unfortunately, due to the lack of diplotype combinations of haplotypes A3, A5, A6, and A7, their relative effects were not determined.

The highest and the lowest Diam values were seen in diplotypes A7A4 (3.7 cm) and A7A8 (2.8 cm), with a ~ 0.90 cm difference between them (**Figure 3A**).

The pedigree map showed that ‘Little Chief’ was the only source of A1, while both A2 and A3 came from ‘Red Fairy’. A4 and A5 were inherited from ‘Violette’ and *R. wichuraiana*, respectively (**Figure 3A**). However, A6 and A7 appeared to have arisen from recombination events between the parents of WOB26 (*R. wichuraiana* and ‘Old Blush’). Lastly, ‘Little Chief’, a very small flowered miniature rose, was the source of A8 (*q-*allele).

**Figure 4:**
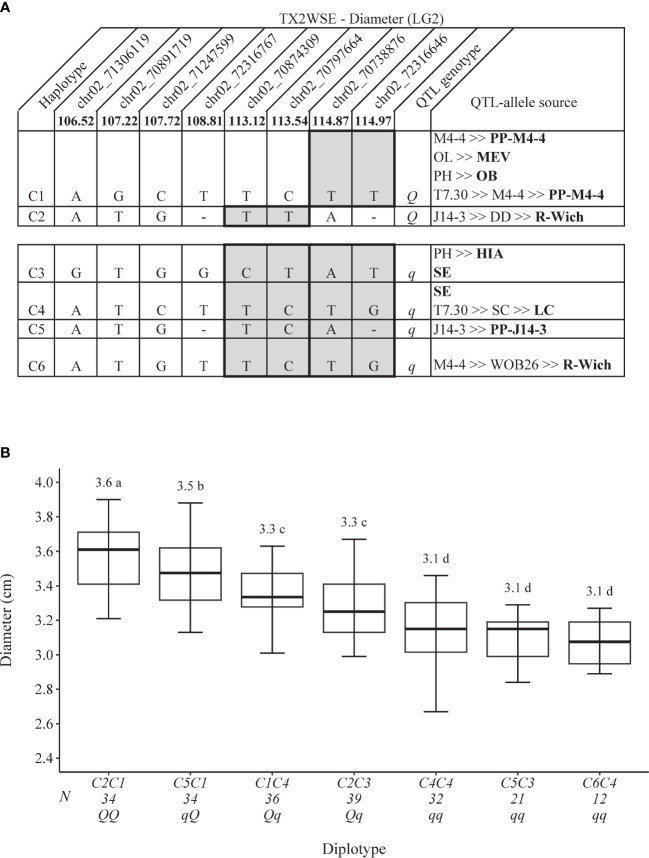
QTL genotypes for diameter at *q*Diam.TX2WSE-LG2 for diploid rose breeding parents with haplotype names, SNP sequences, origin sources, and alleles for predictive SNP markers associated with *Q-* or *q*-alleles for increasing or decreasing the diameter, respectively, are shaded **(A)** and the diplotype effect of the most common haplotypes associated with the diameter at *q*Diam.TX2WSE-LG2 **(B)**. Means not connected by the same letter are significantly different (*p*<0.05) within each population using the non-parametric multiple comparison Steel–Dwass test. N = diplotype sample size. OL, ‘Ole’; MEV, ‘M Nathalie Nypels’; PH, ‘Papa Hemeray’; OB, ‘Old Blush’; T7-30, TAMU7-30; R-Wich, *Rosa wichuraiana*; HIA, ‘Hiawatha’ SE, ‘Srdce Europy’; SC, ‘Sweet Chariot’; LC, ‘Little Chief’.

The LG2 QTLs, *q*Diam.TX2WOB-LG2.2 and *q*Diam.TX2WSE-LG2, passed our inclusion threshold and underwent haplotype analysis, as they were detected at the same genomic region across both populations with strong evidence and PVE up to 16%. Interestingly, the end of the QTL interval was defined at the same physical position (72,316,646 bp) in both consensus maps (**Figures 3C**, **4A**).

For the TX2WOB population, 10 SNPs between 81.4 and 91.4 cM spanning ~10 cM (~2.1 Mbp) in the *q*Diam.TX2WOB-LG2.2 were selected for the haplotype analysis (**Figure 3C**). Six distinct SNP haplotypes were identified, of which B1 through B5 were associated with increasing Diam and assigned to the *Q-*allele. B6, the haplotype linked to decreasing this trait, was designated the *q-*allele (**Figure 3C**). B5 and B6 were the most prevalent haplotypes (**Figure 3D**).

The haplotype/diplotype effects showed that B1 had a greater effect than B2 when comparing the B1B5 to B2B5 and B1B6 to B2B6 diplotypes. Meanwhile, B2 showed an equal effect as B3 (B2B5 to B3B5, and B2B6 to B3B6) (**Figure 3D**). B5 had a greater effect on increasing Diam than B6 (B2B5 to B2B6, B1B5 to B1B6, and B3B5 to B3B6) and B4 (B2B5 to B2B4). Lastly, B4 had more magnitude in increasing Diam than B6 (B2B4 to B2B6). Hence, these results indicated multiple *Q-*alleles with different effects at this locus, in which B1 had the largest effect (*Q_1_*), followed by B2 and B3 (*Q_2_*). However, the lack of representation of all diplotype combinations hampered the estimation of B4 and B5 effects compared to other haplotypes (**Figure 3D**). At this locus, the highest and the lowest diplotype means were observed in individuals with the B1B5 (~3.7 cm) and B3B6 (~3.3) diplotypes, respectively, with a ~ 0.40 cm difference between them.

Based on pedigree information, some parents shared identical haplotypes in this study, although they were inherited from different ancestors (**Figure 3C**). B1 was identical by state but not identical by descent, as it came from ‘Old Blush’ through J4-6 and M4-4 parents or from ‘Violette’ through ‘Vineyard Song’. Likewise, the B3 was traced to ‘Violette’ and ‘PP-J14-3-2’ through ‘SC’ and J14-3, respectively. B2 was inherited from *R. wichuraiana* through J14-3, whereas B4 and B5 came from ‘Little Chief’ and ‘Red Fairy’, respectively. ‘Little Chief’ and ‘Red Fairy’ were the sources of B6 (*q*-allele).

As for TX2WSE, *q*Diam.TX2WSE-LG2 had six unique haplotypes defined with eight SNPs spanning ~8.5 cM (~1 Mbp) (**Figure 4A**). C1 and C2 were associated with increasing the trait and were assigned to the *Q-*allele, while C3–C6 were the haplotype related to decreasing Diam and assigned to *q-*allele. The estimation of diplotype effects revealed that C1 (*Q-*allele) appeared to lead to a greater Diam than C3 (*q-*allele) based on two diplotype combinations (C5C1 to C5C3 and C2C1 to C2C3) (**Figure 4B**). The same was true for C2 and C5 when comparing C2C1 to C5C1 and C2C3 to C5C3 diplotypes. In addition, C1 had a greater effect on increasing Diam than C4 (C1C4 to C4C4). At the same time, C4 and C6 showed a similar magnitude in lowering Diam (C4C4 to C6C4). The difference between the highest (C2C1) and the lowest (C6C4) diplotype at this locus was ~ 0.50 cm (**Figure 4B**).

The pedigree map showed that *R. wichuraiana* was the only source of C2, while C1 came from different sources, ‘Old Blush’, PP-M4-4, or MEV (**Figure 4A**). Similarly, C3 was inherited from HIA or ‘Sweet Chariot’, C4 from ‘Sweet Chariot’ or ‘Little Chief’, C5 from PP-J14-3, and C6 from *R. wichuraiana*.

#### Flower dry/fresh weight

**Figure 5:**
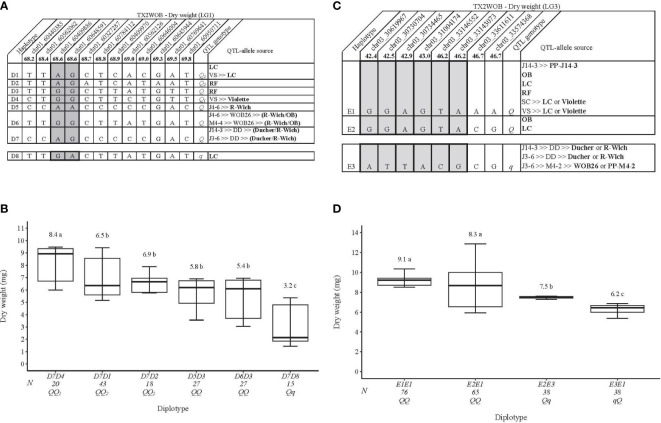
QTL genotypes for dry weight in diploid rose breeding parents with haplotype names, SNP sequences, origin sources, and alleles for predictive SNP markers associated with *Q-* or *q*-alleles for increasing or decreasing the dry weight, respectively, are shaded **(A, C)**, and the diplotype effect of the most common haplotypes associated with the dry weight **(B, D)** at *q*DWT.TX2WOB-LG1 and *q*DWT.TX2WOB-LG3, respectively. The *Q* without a subscript indicates that were not able to categorize this haplotype due to the lack of appropriate diplotype combinations. Means not connected by the same letter are significantly different (*p*<0.05) within each population using the non-parametric multiple comparison Steel–Dwass test. N = diplotype sample size. LC, ‘Little Chief’; VS, ‘Vineyard Song’; RF, ‘Red Fairy’; OB, ‘Old Blush’; SC, ‘Sweet Chariot’; R-Wich, *Rosa wichuraiana*.

For TX2WOB, on the LG1 QTL, *q*DWT.TX2WOB-LG1, eight distinct SNP haplotypes (D1–D8) were identified using 12 SNPs within the QTL interval (68.2–69.8 cM) spanning ~0.5 Mbp on the *Rosa chinensis* genome v1.0 (Hibrand Saint-Oyant et al., 2018) (**Figure 5A**). D8 was the only haplotype associated with decreasing DWT and was assigned to the *q-*allele, while D1–D7 haplotypes were associated with increasing the trait (*Q-*allele) (**Figure 5A**). The assessment of haplotype effects showed multiple QTL alleles of different effects at this locus. Thus, haplotype effects order was D4>[D1=D2]>D8 corresponding to *Q*_1_, *Q*_2_, and *q*, respectively, based on comparisons among D7D4, D7D1, D7D2, and D7D8 diplotypes. In addition, D5 and D6 (*Q-*alleles) had similar magnitudes in increasing DWT (**Figure 5B**). However, we could not determine their relative effects among other haplotypes due to the low representation of various compound diplotypes. The same was true for D3 and D7 (*Q-*alleles). The DWT difference was ~5 mg between the highest (D7D4, 8.4 mg) and the lowest (D7D8, 3.2 mg) diplotypes (**Figure 5B**). The pedigree records showed that ‘Little Chief’ was the source of D1, ‘Red Fairy’ for D2 and D3, D4 for ‘Violette’, and D5 for *R. wichuraiana* (**Figure 5A**). D6 appeared to have arisen from recombination events between the parents of WOB26 (*R. wichuraiana* and ‘Old Blush’), and D7 originated from a recombination event between the parents of DD (‘Ducher’ and *R. wichuraiana*). ‘Little Chief’ was the source of D8 (*q-*allele).

Regarding the LG3 DWT QTL, eight SNP markers in *q*DWT.TX2WOB-LG3 (42.4–46.7 cM) spanning ~3 cM were selected for haplotype analysis (**Figure 5C**). Three distinct SNP haplotypes were identified, in which E1 was the most common haplotype (**Figures 5C, D**). Haplotypes E1 and E2 were linked to high DWT and assigned to the *Q-*allele, while E3 was the only haplotype related to lowering (*q-*allele) (**Figure 5C**). The estimation of diplotype effects indicated that E1 and E2 had a larger effect than E3 when comparing the E2E1 to E2E3 and E2E1 to E3E1, respectively (**Figure 5D**). Similar magnitudes in increasing DWT were registered between E1 and E2 based on E1E1 to E2E1. Overall, E1E1(*QQ*) and E3E1(*qQ*) showed the highest (~9.1 mg) and the lowest (6.2 mg) DWT, respectively.

According to the pedigree information, some parents were identical by state (IBS), not identical by descent (IBD). E1 was inherited from five sources (IBS), namely, PP-J14-3, ‘Old Blush’, ‘Little Chief’, ‘Red Fairy’, or ‘Violette’ (**Figure 5C**). The source of E2 was either ‘Old Blush’ or ‘Little Chief’, while E3 came from ‘Ducher’, *R. wichuraiana*, WOB26, or PP-M4-2.

**Figure 6:**
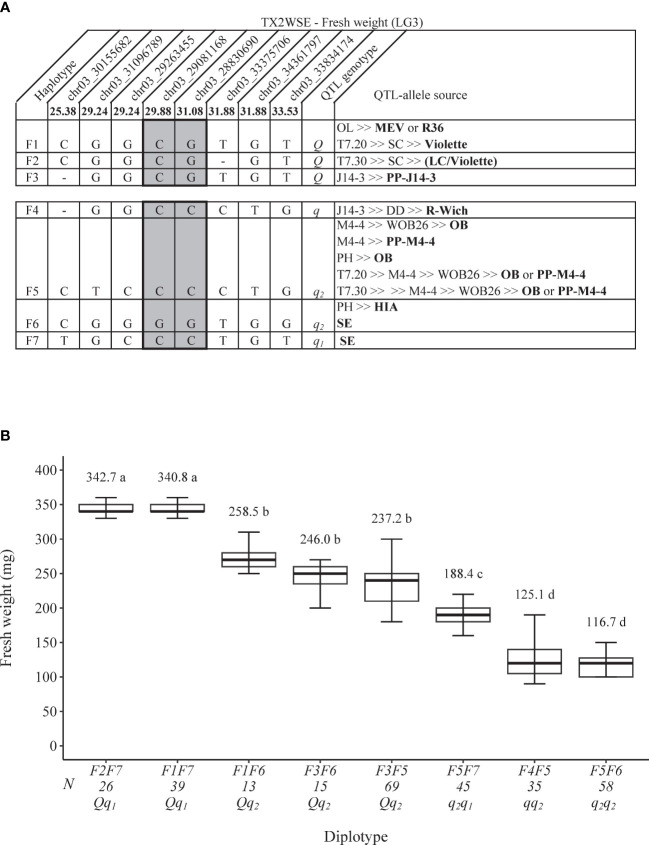
QTL genotypes for fresh weight at *q*FWT.TX2WSE-LG3 for diploid rose breeding parents with haplotype names, SNP sequences, origin sources, and alleles for predictive SNP markers associated with *Q-* or *q*-alleles for increasing or decreasing the fresh weight, respectively, are shaded **(A)**, and the diplotype effect of the most common haplotypes associated with the fresh weight at *q*FWT.TX2WSE-LG3 **(B)**. Means not connected by the same letter are significantly different (*p*<0.05) within each population using the nonparametric multiple comparison Steel–Dwass test. N = diplotype sample size. OL, ‘Ole’; MEV, ‘M Nathalie Nypels’; T7-20, TAMU7-20; SC, ‘Sweet Chariot’; T7-30, TAMU7-30; LC, ‘Little Chief’; R-Wich, *Rosa wichuraiana*; OB, ‘Old Blush’; PH, ‘Papa Hemeray’; HIA, ‘Hiawatha’; SE, ‘Srdce Europy’.

As for FWT QTL, seven SNP haplotypes (F1–F7) were identified using eight SNPs within the *q*FWT.TX2WSE-LG3 region [25.38–33.53 cM) spanning ~3.7 Mbp on the *Rosa chinensis* genome v1.0 (Hibrand Saint-Oyant et al., 2018)] (**Figure 6A**). F1, F2, and F3 were the haplotypes associated with increasing FWT and were assigned to the *Q-*allele. Conversely, F4, F5, F6, and F7 were associated with decreasing the trait (*q-*allele) (**Figure 6A**). All *Q*-alleles (F1, F2, and F3) had an equal effect when comparing F2F7 to F1F7 and F1F6 to F3F6 diplotypes (**Figure 6B**). In addition, no difference was observed between F5 and F6 (F3F6 to F3F5). The estimation of diplotype effects revealed that F7 led to greater FWT than F6 (F1F7 to F1F6 and F5F7 to F5F6), and F3 had more effect than F4 and F5 (F3F5 to F4F5 and F3F6 to F5F6). The same was true for F1 and F5 when comparing F1F7 to F5F7 and F1F6 to F5F6. Thus, *q*-alleles with different effects on decreasing FWT were identified at this locus and coined *q*_1_ (F7) and *q*_2_ (F5 = F6). The difference between the highest (F2F7) and lowest (F5F6) diplotypes was ~226 mg.

The pedigree map showed that MEV, R36, or ‘Violette’ were F1 sources, PP-J14-3 was for F3, while F2 appeared to have arisen from recombination events between the parents of ‘SC’ (‘LC and ‘Violette’) (**Figure 6A**). F4 came from one source (*R. wichuraiana*), whereas F5 originated from two sources (‘Old Blush’ and PP-M4-4). Lastly, F6 was inherited from HIA or ‘Sweet Chariot’, and the latter was the only source of F7.

In this study, the interplay between major QTLs (*q*DWT.TX2WOB-LG1 and *q*DWT.TX2WOB-LG3) for flower dry weight in TX2WOB was studied through the compound LG1/LG3 QTL genotype to differentiate between the effect of these QTLs (**Supplementary Figure 17**). The results showed two copies of *QQ* alleles at *q*DWT.TX2WOB-LG1 tended to have a higher DWT than those at *q*DWT.TX2WOB-LG3 (**Supplementary Figure 17**). Likewise, one dose of *q-*allele at *q*DWT.TX2WOB-LG1 showed more effect than one copy of *q-*allele at *q*DWT.TX2WOB-LG3. This finding also coincided with the diplotype effects for this trait, which showed that the difference between the highest and lowest diplotypes was higher at LG1 (5.2 mg) than at LG3 (2.9 mg) (**Figures 5B, D**). Likewise, the analysis of QTL genotypes revealed that at *q*DWT.TX2WOB-LG1 has a larger effect on DWT than *q*DWT.TX2WOB-LG3 (3.2 vs. 1.3 mg) (**Supplementary Figures 14A, B**).

Therefore, this finding might suggest that *q*DWT.TX2WOB-LG1 had more effect of increasing/decreasing the DWT than *q*DWT.TX2WOB-LG3. However, comparisons to offspring with homozygous *q-*alleles at both loci could not be made as such germplasm was absent in the TX2WOB dataset. Moreover, the small sample size of offspring having three *Q-*allele doses (heterozygous at the LG1 and homozygous at the LG3) might cause an underestimation of the effect of the LG3 QTL.

### Number of petals

**Figure 7:**
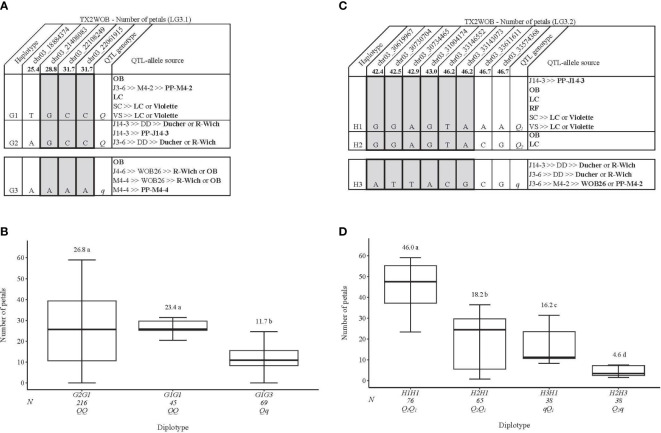
QTL genotypes for number of petals in diploid rose breeding parents with haplotype names, SNP sequences, origin sources, and alleles for predictive SNP markers associated with *Q-* or *q*-alleles for increasing or decreasing the number of petals, respectively, are shaded **(A, C)**, and the diplotype effect of the most common haplotypes associated with the number of petals **(B, D)** at *q*NP.TX2WOB-LG3.1 and *q*NP.TX2WOB-LG3.2, respectively. The *Q* without a subscript indicates that were not able to categorize this haplotype due to the lack of appropriate diplotype combinations. Means not connected by the same letter are significantly different (*p*<0.05) within each population using the nonparametric multiple comparison Steel–Dwass test. N = diplotype sample size. OB, ‘Old Blush’; LC, ‘Little Chief’; SC, ‘Sweet Chariot’; VS, ‘Vineyard Song’; R-Wich, *Rosa wichuraiana*; RF, ‘Red Fairy’.

This analysis considered two major QTLs that were mapped on LG3 in TX2WOB, which showed high PVE and posterior intensity. The four SNPs (25.4–31.7cM) spanning ~3.2 Mbp in the *q*NP.TX2WOB-LG3.1 region were chosen for haplotyping (**Figure 7A**). Three SNP haplotypes were discovered in which G1 and G2 were associated with increasing NP and assigned to the *Q-*allele, while G3 was linked to decreasing the trait (*q-*allele) (**Figure 7A**). G2G1 was the most common diplotype (216 individuals) (**Figure 7B**). The analysis of the haplotype effects showed that the effects of G1 and G2 could not be differentiated based on G2G1 and G1G1. Meanwhile, G1 showed a greater effect than G3 (G1G1 to G1G3). The pedigree information indicated that the three haplotypes were IBS. G1 was inherited from ‘Old Blush’, PP-M4-2 ‘Little Chief’, or ‘Violette’ (**Figure 7A**); G2 was from PP-J14-3, ‘Ducher’, or *R. wichuraiana*; and G3 came from PP-M4-4, ‘Old Blush’, or *R. wichuraiana*.

For *q*NP.TX2WOB-LG3.2, eight SNPs (42.4–46.7cM) spanning ~3 Mbp in the QTL region were selected for the analysis (**Figure 7C**). Three SNP haplotypes were detected. H1 and H2 increased NP (*Q-*allele), while H3 was linked to decreasing the trait (*q-*allele). H1 was the most common haplotype (**Figures 7C, D**). The diplotype analysis revealed the presence of three QTL-alleles with different effects on NP and ordered as *Q*_1_ (H1) > *Q*_2_ (H2) > *q* (H3). H1 was inherited from various sources (PP-J14-3, ‘Old Blush’, ‘Little Chief’, ‘Red Fairy’, or ‘Violette’) (**Figure 7C**), H2 came from ‘Old Blush’ or ‘Little Chief’, and H3 originated from ‘Ducher’, *R. wichuraiana*, WOB26, or PP-M4-2.

**Figure 8:**
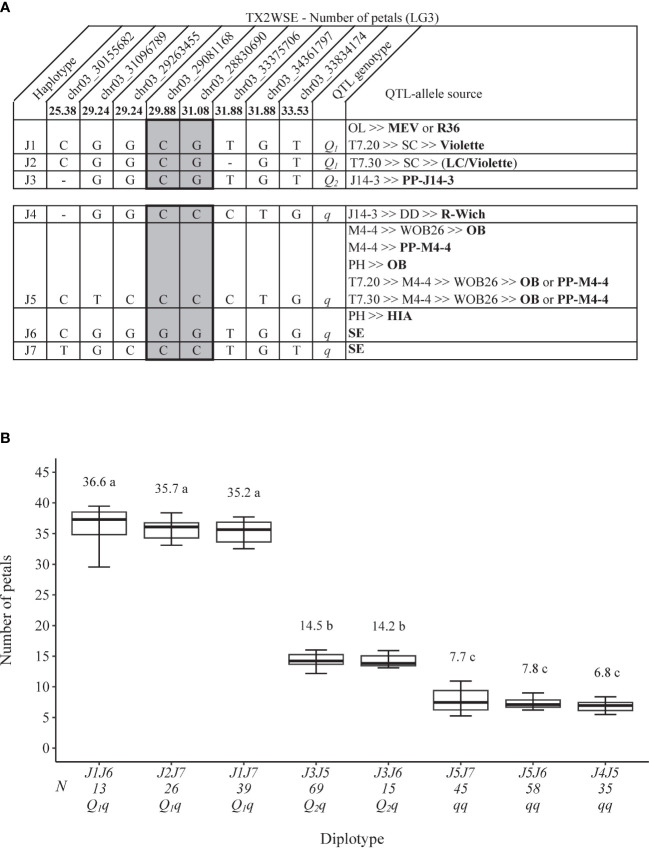
QTL genotypes for number of petals at *q*NP.TX2WSE-LG3 for diploid rose breeding parents with haplotype names, SNP sequences, origin sources, and alleles for predictive SNP markers associated with *Q-* or *q*-alleles for increasing or decreasing the number of petals, respectively, are shaded **(A)**, and the diplotype effect of the most common haplotypes associated with the number of petals at *q*NP.TX2WSE-LG3 **(B)**. The *Q* without a subscript indicates that were not able to categorize this haplotype due to the lack of appropriate diplotype combinations. Means not connected by the same letter are significantly different (*p*<0.05) within each population using the non-parametric multiple comparison Steel–Dwass test. N = diplotype sample size. OL, ‘Ole’; MEV, ‘M Nathalie Nypels’; T7-20, TAMU7-20; SC, ‘Sweet Chariot’; T7-30, TAMU7-30; LC, ‘Little Chief’; R-Wich, *Rosa wichuraiana*; OB, ‘Old Blush’; PH, ‘Papa Hemeray’; HIA, ‘Hiawatha’; SE, ‘Srdce Europy’.

As for TX2WSE, eight SNPs were also chosen to conduct haplotype analysis in the *q*NP.TX2WSE-LG3 region (25.38–33.53 cM) spanning ~3.7 Mbp (**Figure 8A**). Seven SNP haplotypes were identified, in which J1, J2, and J3 were associated with the increasing NP and were given *Q-*alleles. J4, J5, J6, and J7 were related to decreasing the trait (*q-*allele) (**Figure 8A**). The diplotype analysis revealed that J1 and J2 (*Q-*alleles) had a similar effect when comparing J2J7 to J1J7 (**Figure 8B**). No difference was observed between J5 and J6 (J3J5 to J3J6) or between J7 and J6 (J1J7 to J1J6 and J5J7 to J5J6). J1 had a greater effect than J3 (J1J6 to J3J6), and the latter also had a greater effect than J4 and J5 (J3J5 to J4J5 and J3J6 to J5J6). Thus, three QTL alleles of different effects were identified at this locus. The haplotype effects order was [J1 =J2]>J3>[J5=J6=J7] corresponding to *Q*_1_, *Q*_2_, and *q*, respectively. However, the under-representation of some diplotype compounds hindered our ability to conclude the magnitude of the J4 (*q-*allele) effect compared to other haplotypes associated with *q-*alleles (**Figure 8B**). The highest and lowest diplotypes were observed in J1J6 (36.6) and J4J5 (6.8).

The sources of J1 were MEV, R36, or ‘Violette’, J3 was PP-J14-3, while J2 was a recombinant haplotype and originated from recombination events between the parents of ‘SC’ (‘LC and ‘Violette’) (**Figure 8A**). J4 came from *R. wichuraiana*, J5 from ‘Old Blush’ and PP-M4-4, J6 from HIA or ‘Sweet Chariot’, and J7 from ‘Sweet Chariot’.

In addition, the interplay between *q*NP.TX2WOB-LG3.1 and *q*NP.TX2WOB-LG3.2 was assessed (**Supplementary Figure 18**). The analysis revealed one dose of either *Q*- or *q*-alleles at the *q*NP.TX2WOB-LG3.2 increased/decreased NP more than those at *q*NP.TX2WOB-LG3.1 (**Supplementary Figure 18**). This indicates that *q*NP.TX2WOB-LG3.2 has a larger effect than *q*NP.TX2WOB-LG3.1. This result was supported by diplotype effects (~41 vs. ~15) (**Figures 7B, D**) and QTL genotype (~18 vs. 15) (**Supplementary Figures 15A, B**) analyses for *q*NP.TX2WOB-LG3.2 and *q*NP.TX2WOB-LG3.1, respectively. The effect of *qq-*genotypes at both loci could not be determined for the same reason mentioned above. Accordingly, future QTL mapping studies using broader and more diverse germplasm are crucial to enhance the representation of three QTL genotype classes/diplotype combinations.

### Number of petaloids

**Figure 9:**
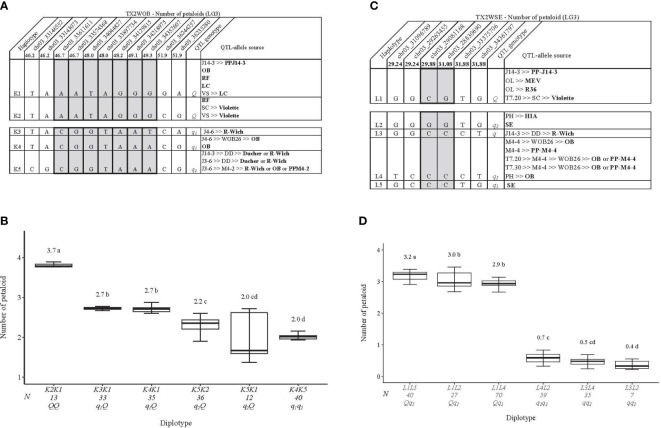
QTL genotypes for number of petaloids in diploid rose breeding parents with haplotype names, SNP sequences, origin sources, and alleles for predictive SNP markers associated with *Q-* or *q*-alleles for increasing or decreasing the number of petaloids, respectively, are shaded **(A, C)**, and the diplotype effect of the most common haplotypes associated with the number of petaloids **(B, D)** at *q*PD.TX2WOB-LG3 and *q*PD.TX2WSE-LG3, respectively. The *Q* without a subscript indicates that were not able to categorize this haplotype due to the lack of appropriate diplotype combinations. Means not connected by the same letter are significantly different (*p*<0.05) within each population using the nonparametric multiple comparison Steel–Dwass test. N = diplotype sample size. OB, ‘Old Blush’; RF, ‘Red Fairy’; LC, ‘Little Chief’; VS, ‘Vineyard Song’; R-Wich, *Rosa wichuraiana*; SC, ‘Sweet Chariot’; OL, ‘Ole’; MEV, ‘M Nathalie Nypels’; T7-20, TAMU7-20; PH, ‘Papa Hemeray’; HIA, ‘Hiawatha’; T7-30, TAMU7-30; SE, ‘Srdce Europy’.

Haplotype analysis was conducted on the LG3 PD QTL mapped in each population. In the TX2WOB population, five SNP markers between 46.2 and 51.9 cM spanning ~3.0 Mbp in the *q*PD.TX2WOB-LG3 were selected for haplotype analysis (**Figure 9A**). Five distinct SNP haplotypes were identified, of which K1 and K2 were associated with increasing PD (*Q-*allele), while K3, K4, and K5 were linked to decreasing PD (*q-*allele) (**Figure 9A**). The assessment of the haplotype/diplotype effects showed the effect of K1 seemed to be similar to that of K2 (K5K2 to K5K1) (**Figure 9B**). Likewise, K3 had the same effect as K4 (K3K1 to K4K1). K2 had a greater effect on increasing PD than K3 by comparing K2K1 to K3K1. In addition, K4 increased PD more than K5 (K4K1 to K5K1). Thus, the haplotypes were ordered from higher to lower effects, [K1=K2]>[K3=K4]>K5 and were assigned the *Q*, *q*_1_, and *q*_2_ QTL alleles, respectively.

K2K1 showed the highest PD (~3.7), K4K5 had the lowest (~2.0), and K1 was the most prevalent haplotype (**Figure 9B**). K1 was traced back to various sources (PP-J14-3, ‘Old Blush’, ‘Red Fairy’, or ‘Little Chief’) (**Figure 9A**). K2 came from ‘Red Fairy’ and ‘Violette’, K3 came from *R. wichuraiana*, K4 from ‘Old Blush’, while K5 was inherited from ‘Ducher’, ‘R-Wich ‘Old Blush’, or PP-M4-2.

In the TX2WSE population, six SNP markers between 29.24 and 31.88 cM spanning ~3.3 Mbp were used in the haplotype analysis of *q*PD.TX2WSE-LG3. Five distinct SNP haplotypes were identified (**Figure 9C**). L1 was the only haplotype associated with increasing PD (*Q-*allele), and L2, L3, L4, and L5 were associated with decreasing the trait (*q-*allele). The results also showed that L1 had a larger effect than L3 and L4 based on L1L2 to L3L2, L1L4 to L3L4, and L1L2 to L4L2 diplotypes (**Figure 9D**). L2 and L4 (*q-*alleles) had a similar effect when comparing L1L2 to L1L4 and L3L4 to L3L2. Likewise, L5 increased PD more than L2 (L1L5 to L1L2). Thus, there were two QTL alleles of different effects associated with decreasing PD, *q*_1_ (L5) > *q*_2_ (L2=L4). However, the effect of L3 (*q-*allele) was not determined due to the low representation of some diplotype combinations (**Figure 9D**).

The difference between the highest (L1L5) and lowest (L3L2) was ~3 petaloids. The pedigree map showed that L1 was inherited from PP-J14-3, MEV, R36, and ‘Violette’ (**Figure 9C**). The source of L2 was either ‘Sweet Chariot’ or HIA, while L3 and L5 came from *R. wichuraiana* and ‘Sweet Chariot’, respectively. Lastly, ‘Old Blush’ and PP-M4-4 were the sources for L4.

## Discussion

### Heritability and G×E interactions

In this study, narrow sense heritability was low to moderate for Diam, consistent with the prior studies (Liang et al., 2017a; Liang et al., 2017b). In addition, the moderate narrow sense heritability estimated for DWT and FWT agreed with those previously reported for DWT (Liang et al., 2017a; Liang et al., 2017b). Regarding NP, moderate to moderately high narrow sense heritability was found in this and previous studies (Gitonga et al., 2014; Liang et al., 2017a; Liang et al., 2017b). Lastly, low to moderate narrow sense heritability was observed for PD.

Moreover, all flower size traits, Diam, DWT, and NP showed a moderately high to high broad sense heritability (0.75, 0.82, and 0.87) (**Supplementary Table 6**) as previously reported (Gitonga et al., 2014; Liang et al., 2017a; Liang et al., 2017b). This study highlighted the important additive and non-additive genetic components for the studied traits.

The G×E interaction was varied from low ( $\sigma_{\text{g}\times \text{e}}^{2}/\sigma_{\text{g}}^{2}$ ratio, 0.43–0.65) for NP and DWT to moderate ( $\sigma_{\text{g}\times \text{e}}^{2}/\sigma_{\text{g}}^{2}$ ratio, 1.00) for Diam. These results indicate that DWT and NP have a strong genetic component with less environmental influence than Diam (Gitonga et al., 2014; Liang et al., 2017a), and hence, the selection for these two traits can be made irrespective of seasons.

The research conclusively showed that all examined traits, over the two evaluated years, experienced a significant reduction when subjected to heat stress (average summer monthly temperature, approximately 28–28.6°C). However, these traits increased under cooler conditions (average spring and fall monthly temperature, approximately 20–22°C) (**Supplementary Table 2**; **Supplementary Figure 3**). This aligns with the findings of previous studies (Gitonga et al., 2014; Greyvenstein et al., 2014; Liang et al., 2017a; Liang et al., 2017b).

### Correlations between the flower size traits

DWT was moderately positively correlated with Diam and petal number over all data sets in both populations (**Supplementary Table 8**), as previously reported (Liang et al., 2017b). Therefore, this suggests that the increase in DWT appears to be due to an increase in Diam and/or NP.

In contrast, the correlation between the Diam and the petal number was low in this diploid (Liang et al., 2017b) and in tetraploid rose populations (Yu et al., 2021), indicating that these traits are inherited independently as reflected in the major QTLs for NP and Diam being on different LGs. Furthermore, a high positive correlation between NP and PD in both populations was anticipated as both traits result from transformations from stamens (Dubois et al., 2010).

### QTL detection

#### Flower diameter

In total, 12 QTLs were mapped for Diam distributed over the seven LGs, in which nine QTLs were found in TX2WOB (five data sets) while three QTLs were in TX2WSE (one data set). This finding supports the polygenic nature of Diam in roses, as recently reported in tetraploid roses (Yu et al., 2021), which also mapped several QTLs for Diam on LGs 2, 4, and 7. In addition, finding more QTLs associated with Diam in TX2WOB than in the TX2WSE population may be attributed to the possibility that the genetic background of the two populations is a contributing factor to the differences in the number of QTLs. Moreover, the mean diameter and number of records for TX2WSE were lower than TX2WOB, which also could be another reason that fewer QTLs were detected segregating in the TX2WSE population.

In this study, the LG1 QTL (*q*Diam.TX2WOB-LG1) exhibited a large effect (PVE up to 80%) in TX2WOB and was consistently detected at the distal end of LG1 (60.44–60.95 Mbp) in the cool seasons (average temperature of spring and fall, ~20 to 22°C) and mean 2015 but not during the warm seasons of 2015 and 2021 (average temperature of summer, ~28–28.6°C) (**Supplementary Table 2**; **Supplementary Figure 3**). Most other QTLs identified for Diam were detected in either the summer of 2015 or 2021. The differential expression of these QTLs in the warmer season may be, in part, responsible for the significant G×E interaction seen in this and previous studies of this germplasm (Liang et al., 2017a; Liang et al., 2017b). The GGE biplot clearly shows that the vector of summer 2015 is very different from those of the cooler seasons (**Supplementary Figure 7**).

Of the minor QTLs identified, the population-specific QTL LG2, *q*Diam.TX2WOB-LG2.1 overlapped with QTL previously (qfdia-2-2) described in different germplasm (tetraploid rose) for Diam with PVE ~12% (Yu et al., 2021). Two pairs of minor QTLs on LG2 (*q*Diam.TX2WOB-LG2.2 and *q*Diam.TX2WSE-LG2) and LG3 (*q*Diam.TX2WOB-LG3.2 and *q*Diam.TX2WSE-LG3) overlapped and were common between the TX2WOB and the TX2WSE populations. Lastly, the QTL on LG6 (*q*Diam.TX2WOB-LG6.2) was seen in two data sets in 2015.

#### Flower weight

For the first time, four QTLs were mapped for DWT using four data sets of TX2WOB, whereas two QTLs were detected for FWT using one data set for each population. Thus, this study indicates that the flower weight is polygenic in roses. Two QTLs with large effects were identified for flower weight in which the QTL on LG1 was only associated with DWT, whereas the QTL on LG3 was common between FWT and DWT (**Table 2**).

*q*DWT.TX2WOB-LG1 showed a large effect (PVE up to 41%) for DWT and was identified at the lower part of LG1 (60.44–60.95 Mbp) over three data sets (spring, fall, and the mean). However, this QTL was not detected in the warmer summer months, thus not pertinent in developing heat-tolerant roses. On the other hand, the LG3 QTL for DWT (*q*DWT.TX2WOB-LG3) and FWT (*q*FWT.TX2WOB-LG3 and *q*FWT.TX2WSE-LG3) was the most environmentally stable, as it consistently mapped between ~ 30.15 and 39.34 Mbp for both populations under warm (average temperature of summer, ~29°C) and cool (average temperature of spring and fall, ~20–22°C) conditions. In addition, this QTL was stable regardless of the two methods used in phenotyping the flower weight (fresh/dry). Previous studies reported that heat stress reduces flower size in roses and diminishes their overall quality and durability, thus lowering their market values (Marissen, 2001; Shin et al., 2001; Wahid et al., 2007). Greyvenstein et al. (2014) found that flower dry weight decreased with increasing growing temperatures in the garden rose cultivars. Therefore, this QTL is important for breeding if warm summers are an issue compared to the LG1 QTL. However, more QTL mapping studies using different and wider germplasm are crucial to validate the major LG1 QTL and the other minor QTLs.

#### Number of petals

The PBA approach identified two major QTLs associated with NP on LG3 and one minor QTL on LG2. The first QTL was between ~30.00 and 33.80 Mbp and consistently appeared across five data sets in TX2WOB (*q*NP.TX2WOB-LG3.2) and the one set data of TX2WSE (*q*NP.TX2WSE-LG3), with PVE, ranged from 34% to 76%. However, in most data sets, this QTL showed high statistical power for a second QTL and QTL posterior intensity >1, indicating that the QTL intervals may harbor two QTLs. This region on LG3 (33.24–33.53 Mbp) was identified as the region for the DOUBLE FLOWER locus (Hibrand Saint-Oyant et al., 2018). In addition, several studies have reported the major QTL for NP located in this region on LG3 (Rajapakse et al., 2001; Crespel et al., 2002; Linde et al., 2006; Hibrand-Saint Oyant et al., 2008; Spiller et al., 2011; Koning-Boucoiran et al., 2012; Roman et al., 2015; Hibrand Saint-Oyant et al., 2018). Furthermore, a recent study has proposed that the APETELA2 gene is involved in determining the number of petals (Hibrand Saint-Oyant et al., 2018).

The other major QTL on LG3, *q*NP.TX2WOB-LG3.1 was identified in the fall and the mean 2015 data sets with PVE up to 37%. This QTL was environment specific, since it was only expressed in the fall season. It may be attributed to factors other than temperatures, such as day length or flowers initiated from different anatomical structures (e.g., primary shoots in spring and secondary/tertiary shoots in fall). This result is supported by the GGE biplot, which showed that the fall season discriminates the genotypes differently than the other two seasons.

Using the DOUBLE FLOWER locus as a co-factor impacted the QTL mapping for TX2WSE, in which *q*NP.TX2WSE-LG3.CF was mapped between 27.80 and 29.08 Mbp with PVE of 41%. Concerning TX2WOB, the co-factor results were consistent with the original analysis, although *q*NP.TX2WOB-LG3.CF had a wider interval (27.65–33.57 Mbp) over three data sets, and it was even wider in the fall and confounded in both *q*NP.TX2WOB-LG3.1 and *q*NP.TX2WOB-LG3.2. Moreover, all co-factor QTLs in both populations showed a lower effect (PVE) than those detected without co-factor analysis. This finding suggests that the co-factor helped identify a new QTL region between 27 and 29 Mbp, which had a minor effect that the DOUBLE FLOWER locus could mask.

This finding coincided with two previous studies using genome-wide association studies. Hibrand Saint-Oyant et al. (2018) mapped two neighboring loci controlling NP on LG3 when NP was analyzed as a qualitative trait (simple versus double flowers), the primary locus was at 33.08 and 33.94 Mbp and overlapped with the DOUBLE FLOWER locus, while the secondary locus was between ~28.00 and 29.00 Mbp. Likewise, two closely linked loci at 29.0 Mb and 33.3 Mbp on LG3 were found to be associated with this trait (Schulz et al., 2021).

In addition, a minor QTL on LG2 appeared twice at two different genomic regions (8.20–26.21 and 53.32–64.33 Mbp) in both QTL mapping analyses (with and without co-factor). These QTLs could be the same as the LG2 QTLs identified earlier by Roman et al. (2015); Bourke et al. (2018), and Hibrand Saint-Oyant et al. (2018).

This study confirms the two tightly linked loci on LG3 (~27.80–33.83 Mbp) control NP in double flowers (Hibrand Saint-Oyant et al., 2018; Schulz et al., 2021). Additionally, our study provides more evidence that the QTL on LG2 had a minor effect and was supported by other studies using diploid and tetraploid rose germplasm. Moreover, the third QTL on LG3 (~18.88–22.06 Mbp) was novel and had not been reported earlier. Further genetic studies are needed to validate this QTL using broader/more diverse germplasm.

### Number of petaloids

FlexQTL software detected one major QTL on LG3 associated with PD that was consistently mapped across both populations. This QTL was between 33.14 and 36.23 Mbp, and 31.09 and 33.83 Mbp for *q*PD.TX2WOB-LG3 and *q*PD.TX2WSE-LG3, respectively, with PVE 17%–41%. To our knowledge, this is the first QTL report for PD.

Therefore, future studies using data over several environments (seasons, years, and locations) from more diverse germplasm are essential to test robust LG3 QTL across environments and identify whole genetic pathways that regulate PD in roses.

### SNP haplotypes, sources, and effects of QTL alleles

Haplotype analyses for the major QTLs and those common among populations revealed several SNP haplotypes and predictive SNP marker(s) associated with increasing/decreasing QTL alleles. In addition, multiple functional alleles with different effects were found for most traits of this study.

According to pedigree information, for Diam, the ancestors ‘Old Blush’ and *R. wichuraiana* from J14-3 were common sources of *Q-*alleles across populations, while ‘Violette’ was the common source of *Q-*allele over LG1 and LG2 loci of TX2WOB. In addition, ‘Violette’ and PP-J14-3 were the common sources for *Q-*alleles for NP, PD, FWT, and DWT over the two populations. Moreover, ‘Little Chief’, a very small diameter flowered rose, was the common source of *q-*allele for Diam across populations, while *R. wichuraiana*, a single (five petals) flowered species rose, through J14-3 was a common source of *q-*allele for most loci associated with NP, PD, FWT, and DWT across populations.

Alternatively, the genetic information of estimated diplotype effects can be utilized for future selection, such as A7A4 (LG1), B1B5 (LG2) for TX2WOB, and C2C1 (LG2) for TX2WSE for larger Diam; regarding flower weight, D7D4 (LG1) and E1E1 (LG3) for TX2WOB, while F2F7 and F1F7 (LG3) for TX2WSE; and NP, G2G1, G1G1, and H1H1of both major QTLs on LG3 for TX2WOB, whereas J1J6, J2J7, and J1J7 for TX2WSE. These results will aid breeders in parental selection to develop cultivars with high ornamental quality, consequently increasing the market value of roses. Eventually, major QTLs for these traits could be used to develop high-throughput DNA tests for routine use in a DNA-informed breeding program.

### QTL co-localization among traits

**Figure 10:**
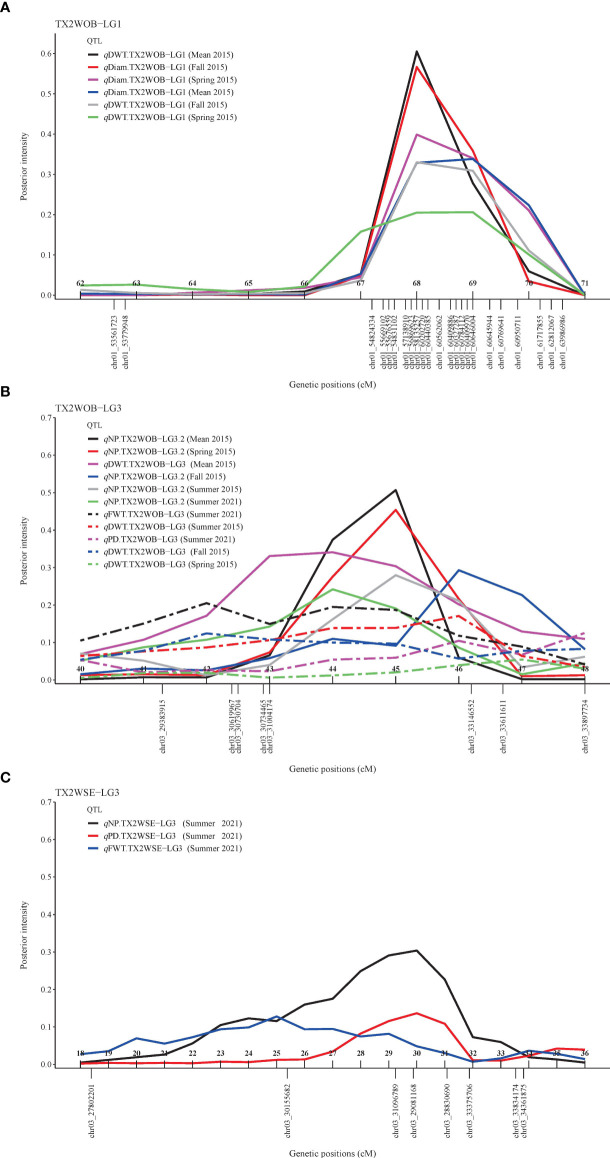
Position of putative QTLs and peaks controlling the diameter (Diam) and dry weight (DWT) in diploid rose at LG1 in TX2WOB **(A)**, DWT, fresh weight (FWT), number of petals (NP), and number of petaloids (PD) at LG3 in TX2WOB **(B)**, and FWT, NP, and PD at LG3 in TX2WSE **(C)** using MapChart software.

In this study, QTLs of flower size traits were clustered at the same genomic region on LG1 and LG3 in the rose genome (**Figure 10**). The first QTL cluster was at the lower part of LG1 (~60.44–60.95 Mbp) for Diam (*q*Diam.TX2WOB-LG1) and DWT (*q*DWT.TX2WOB-LG1) (**Figure 10A**). The haplotype results showed that both loci are in the coupling phase with each other, supported by a positive correlation between the traits.

The genomic region located between 60.44 and 60.95 Mbp on chromosome 1 of the *Rosa chinensis* genome v1.0 (Hibrand Saint-Oyant et al., 2018), according to data derived from the GDR database, encompasses 87 candidate genes (**Supplementary Table 9**). Among those, five genes appear to directly affect plant growth and/or floral development. A number of the cytochrome P450 was reported in this genomic region. A study by Liu et al. (2015) reported CYP715A1 (At5g52400), the lone representative of P450 family in *Arabidopsis thaliana*, underlining its fundamental role in regulating petal expansion, emission of volatiles, and flower maturation. Furthermore, actin-depolymerizing factor 7 (ADF) was found in this region, which is known to be a key regulator of F-actin organization, flowering, and cell and organ expansion in *Arabidopsis*. This F-actin was reported to be required for various cellular processes, including cell division and expansion (Kost et al., 1999). Similarly, a study (Li et al., 2009) on cotton (*Gossypium hirsutum*) reveals that ADF genes (GhADF6 and GhADF8) contribute to petal development and found that the low levels of the ADF genes were detected at the early stages of petal development, while a surge in the gene expression was observed at the later stages of petal development. F-box protein, one of the super protein families, has also been identified in this region. Zhang et al. (2019) pointed out the diverse functionalities of the F-Box genes, including roles in development processes such as plant hormonal signal transduction, floral development, circadian rhythms, responses to biotic and abiotic stresses, and others. The other candidate gene in this QTL interval is FAR1-related sequence 5, which is recognized for its vital role in plant growth and developmental processes, including shoot meristem and floral development in *Arabidopsis* (Li et al., 2016; Ma and Li, 2018). Lastly, two SEUSS-like 2 genes were found in this genomic region on chromosome 1, which was reported to have a role in the negative regulation of the floral homeotic gene AGAMOUS (AG) in *Arabidopsis*, which is responsible for specifying stamen and carpel identity in plants. Franks et al. (2002) suggested that SEUSS mutant causes the partial transformation of floral organs and a mild decrease in the number of floral organs. Furthermore, double mutation of SEUSS and AG led to narrow sepals and petals and reduced plant height.

The second cluster found in TX2WOB was at the middle region of LG3 (~30.61–39.34 Mbp) harbored QTLs for the FWT (*q*FWT.TX2WOB-LG3), DWT (*q*DWT.TX2WOB-LG3), NP (*q*NP.TX2WOB-LG3.2), and PD (*q*PD.TX2WOB-LG3) (**Figure 10B**). Similarly, in TX2WSE, a single cluster of major QTLs was identified for FWT, NP, and PD at the same region in LG3 (~30.15–34.36 Mbp) (**Figure 10C**). In addition, the haplotype results revealed that DWT/FWT and NP shared identical haplotypes associated with increasing/decreasing phenotypic values and their sources, which also partially coincided with PD. Thus, the increase in flower weight seems to be due to an increase in flower diameter and the number of petals or petaloids. This finding was consistent with positive correlations that were found among these traits.

This QTL region on LG3 overlaps with the DOUBLE FLOWER locus (33.24–33.53 Mbp). The candidate gene in this region, APETELA2/TOF (33.23–33.24 Mbp), is proposed to control the switch from simple to double flower and the number of petals within double flowers (Hibrand Saint-Oyant et al., 2018). Moreover, this specific region was previously reported to be associated with the QTL for number of petals (Debener and Mattiesch, 1999; Linde et al., 2006; Koning-Boucoiran et al., 2012; Roman et al., 2015; Bourke et al., 2018; Hibrand Saint-Oyant et al., 2018; Schulz et al., 2021; Yu et al., 2021).

Interestingly, this middle region on LG3 is considered a hot spot in the rose genome as major or candidate genes associated with key morphological traits have been identified such as the continuous flowering locus (RoKSN) (Iwata et al., 2012), and prickle density (TTG2, TESTA TRANSPARENT GLABRA2) (Hibrand Saint-Oyant et al., 2018; Zhou et al., 2020), and QTLs associated with resistance to black spot and cercospora diseases in roses (Rawandoozi et al., 2022; Rawandoozi et al., 2023) have also been reported in this genomic region.

Three additional clusters for minor QTLs were found in TX2WOB. The region between 48.36 and 64.33 Mbp on LG2 was associated with Diam, DWT, and NP. Yu et al. (2021) also mapped the LG2 QTL for Diam (qfdia-2-2), which co-localized with the minor LG2 QTL for Diam (48.36–56.76 Mbp) in this study and NP from another study (Bourke et al., 2018). Schulz et al. (2021) reported two candidate genes that are involved in the regulation of plant growth on LG2 between 65.6 and 68.7 Mbp. The first candidate gene LONGIFOLIA is known to be responsible for regulating cell elongation in *Arabidopsis* (Lee et al., 2006). The second, RhNAC100, was discovered in rose petals, and it was found to be an ethylene-inducible NAC transcription factor. A study showed that the suppression or silencing of RhNAC100 led to a substantial increase in petal size (Pei et al., 2013).

In addition, there was a cluster on LG3 between minor QTL for Diam across both populations (15.44–27.65 Mbp) and the second major QTL for NP (*q*NP.TX2WOB-LG3.1). The distal end of LG5 (85.60–85.70 Mbp) had QTL for DWT and Diam.

Overall, the co-localization of LG1 or LG3 between two or more traits may result from either a single locus with the pleiotropic effect or QTL clusters corresponding to a tight linkage between distinct loci. This has coincided with the positive correlations among the most studied traits.

Further studies of the genetic basis for these flower size traits are needed using larger and more diverse germplasm. In addition, phenotyping in multiple seasons/years is required for Diam, as it showed moderate G×E and petaloids. This would help to test QTL stability across environments and genetic backgrounds before being utilized in a breeding program. Additional work on these populations would be needed to identify the gene(s) responsible for Diam, flower weight, and petaloids.

## Conclusion

This study applied a pedigree-based approach for QTL mapping through FlexQTL software for flower size traits using two multi-parental diploid rose populations phenotyped over six environments in two locations in Texas. Several new and previously reported QTLs associated with NP and Diam were detected. In addition, multiple QTLs were identified for the first time for flower weight and PD.

One major QTL at the middle region on LG3, which may have a pleiotropic effect, was consistently detected across populations and seasons for all traits, excluding Diam. The other major QTL at the distal end of LG1 was specific for Diam and DWT and consistently appeared in 2015 for TX2WOB. In addition, the QTL at the distal end of LG2 for Diam was common across both populations. A novel QTL on LG3 was mapped for the first time between 18.88 and 22.06 Mbp for NP. Using co-factor analysis for NP helped to identify a new QTL ~ 27.00–29.00 Mbp on LG3. This QTL region on LG3 was previously reported to be associated with NP (Hibrand Saint-Oyant et al., 2018; Schulz et al., 2021). In addition, this study revealed a series of QTL alleles of different effects at important loci for most traits. Overall, common sources of *Q-*alleles were ‘Old Blush’ and *R. wichuraiana* from J14-3 for Diam, while ‘Violette’ and PP-J14-3 were for the other flower size traits. The source of *q-*allele was ‘Little Chief’ for Diam and *R. wichuraiana* through J14-3 for the remaining traits.

The estimated diplotype effects can be employed directly in parental selection to improve the ornamental quality of roses. In addition, our study will facilitate using DNA-informed techniques to develop new rose cultivars with high ornamental quality traits.

## Data availability statement

The datasets presented in this study can be found in online repositories. The names of the repository/repositories and accession number(s) can be found below: Genome Database for Rosaceae (http://www.rosaceae.org). The accession number is "tfGDR1072". 

## Author contributions

DB and OR-L conceived and designed this study. ZR and MR performed QTL and haplotype analyses. EY, SL, XW, TH, and QF collected phenotypic data. MY and EY collected tissues and extracted DNA. PK conducted genotyping-by-sequencing and SNP calling. ZR and EY curated genotypic data and produced the linkage maps. ZR wrote the manuscript. The manuscript was reviewed by ZR, EY, SL, XW, QF, TH, MY, MR, PK, DB, and OR-L. DB and OR-L provided project supervision. All authors contributed to the article and approved the submitted version.
