# Supplementary material for: Pedigree-based QTL analysis of flower size traits in two multi-parental diploid rose populations
Source: Front Plant Sci. 2023 Aug 15;14:1226713. doi: 10.3389/fpls.2023.1226713 (PMC10464838; doi:10.3389/fpls.2023.1226713)
Supplement: Supplementary file 1 [file Image_1.pdf]

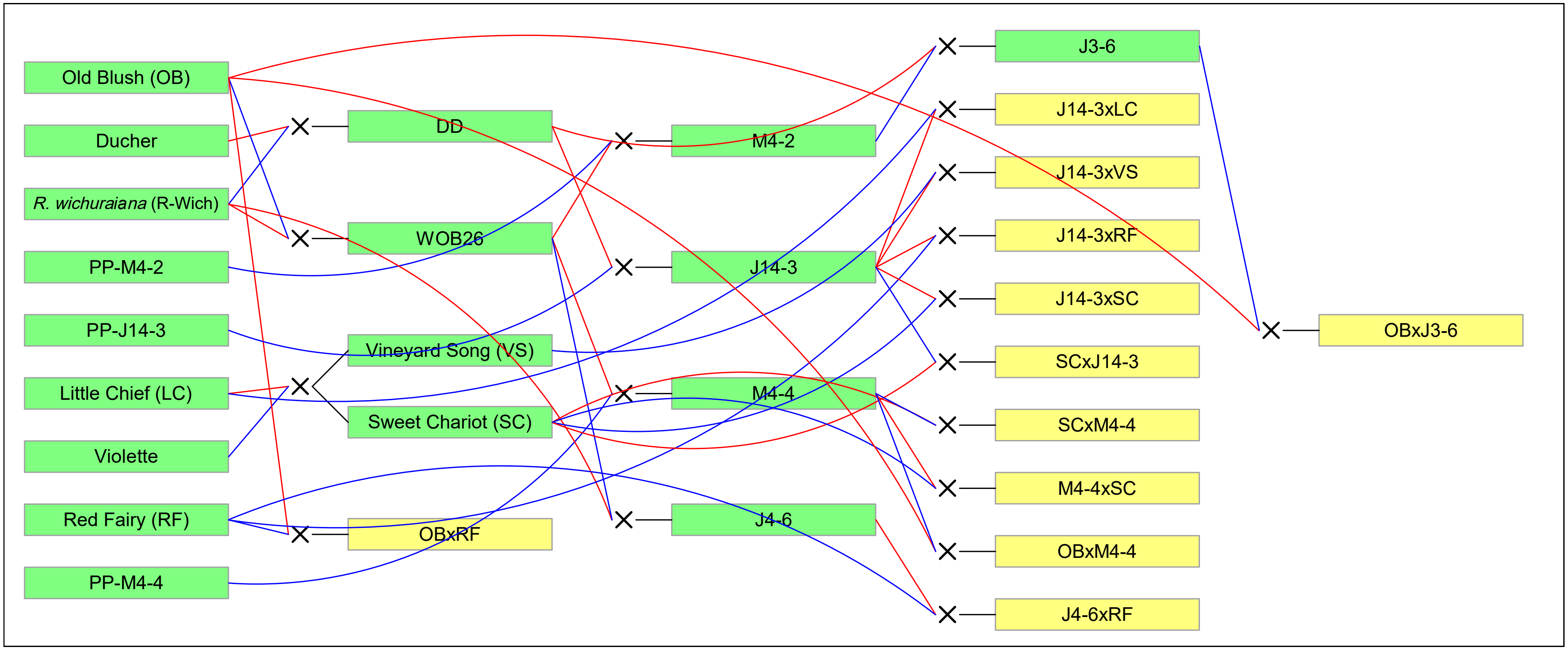

Supplementary Figure 1. Pedigree of the TX2WOB multi-parental population composed of 11 F_1 diploid rose populations derived from intercrossing nine genotypes. Red and blue lines link progeny to female and male parents, respectively, generated using PediMap 1.2.
