# Supplementary material for: Pedigree-based QTL analysis of flower size traits in two multi-parental diploid rose populations
Source: Front Plant Sci. 2023 Aug 15;14:1226713. doi: 10.3389/fpls.2023.1226713 (PMC10464838; doi:10.3389/fpls.2023.1226713)
Supplement: Supplementary file 2 [file Image_2.pdf]

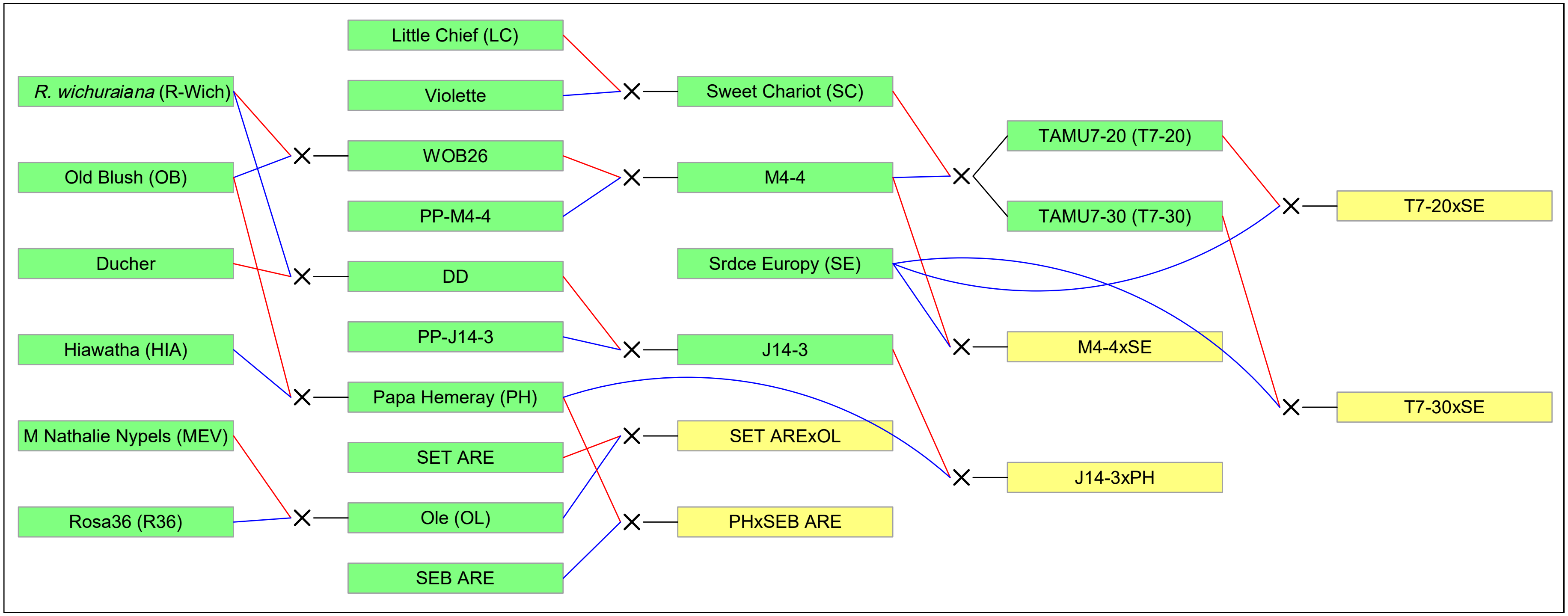

Supplementary Figure 2. Pedigree of the TX2WSE multi-parental population composed of six F_1 diploid rose populations derived from nine parents. Red and blue lines link progeny to female and male parents, respectively, generated using PediMap 1.2.

SET-ARE = *R. setigera*-ARE, SEB-ARE = *R. palustris* f. *plena* EB-ARE.
