# Supplementary material for: Pedigree-based QTL analysis of flower size traits in two multi-parental diploid rose populations
Source: Front Plant Sci. 2023 Aug 15;14:1226713. doi: 10.3389/fpls.2023.1226713 (PMC10464838; doi:10.3389/fpls.2023.1226713)
Supplement: Supplementary file 3 [file Image_3.pdf]

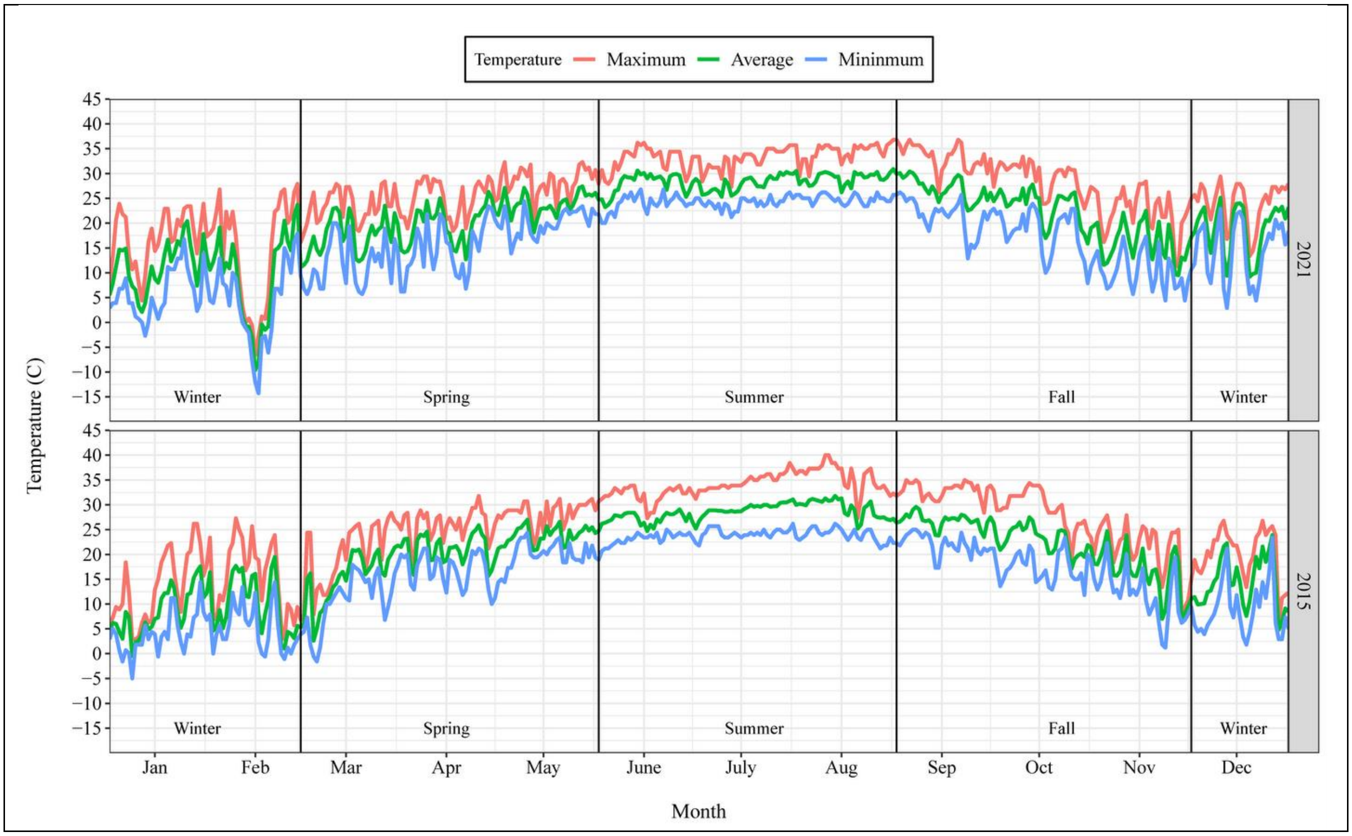

Supplementary Figure 3. Daily maximum, minimum and average temperature in 2015 and 2021 in College Station, Texas.
