# Supplementary material for: Pedigree-based QTL analysis of flower size traits in two multi-parental diploid rose populations
Source: Front Plant Sci. 2023 Aug 15;14:1226713. doi: 10.3389/fpls.2023.1226713 (PMC10464838; doi:10.3389/fpls.2023.1226713)
Supplement: Supplementary file 4 [file Image_4.pdf]

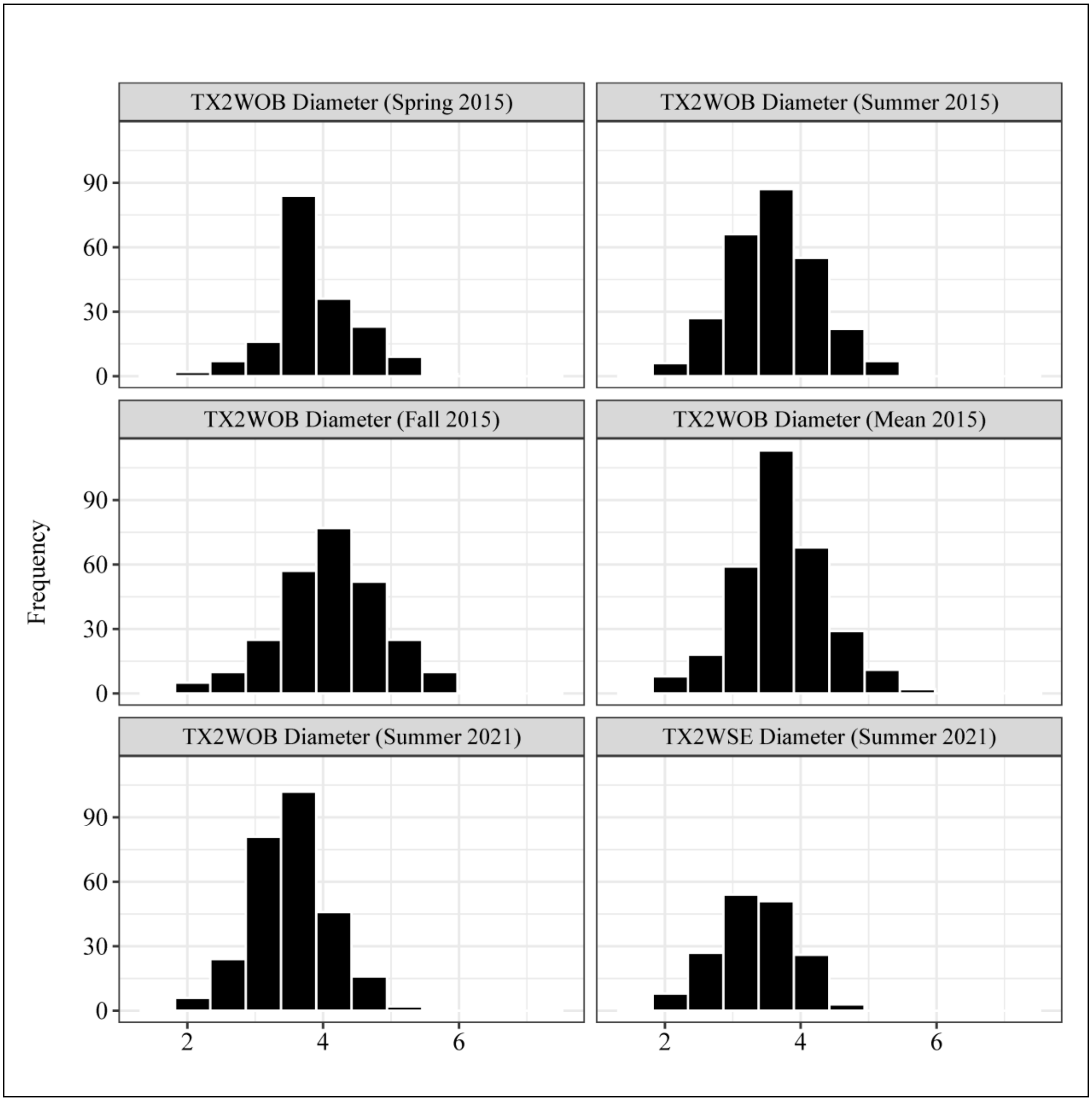

Supplementary Figure 4. Histograms for diameter (Diam) for the TX2WOB diploid rose population phenotyped in Texas in spring, summer, and fall in 2015 in College Station and in summer 2021 in Somerville in TX2WOB and TX2WSE.
