# Supplementary material for: Pedigree-based QTL analysis of flower size traits in two multi-parental diploid rose populations
Source: Front Plant Sci. 2023 Aug 15;14:1226713. doi: 10.3389/fpls.2023.1226713 (PMC10464838; doi:10.3389/fpls.2023.1226713)
Supplement: Supplementary file 5 [file Image_5.pdf]

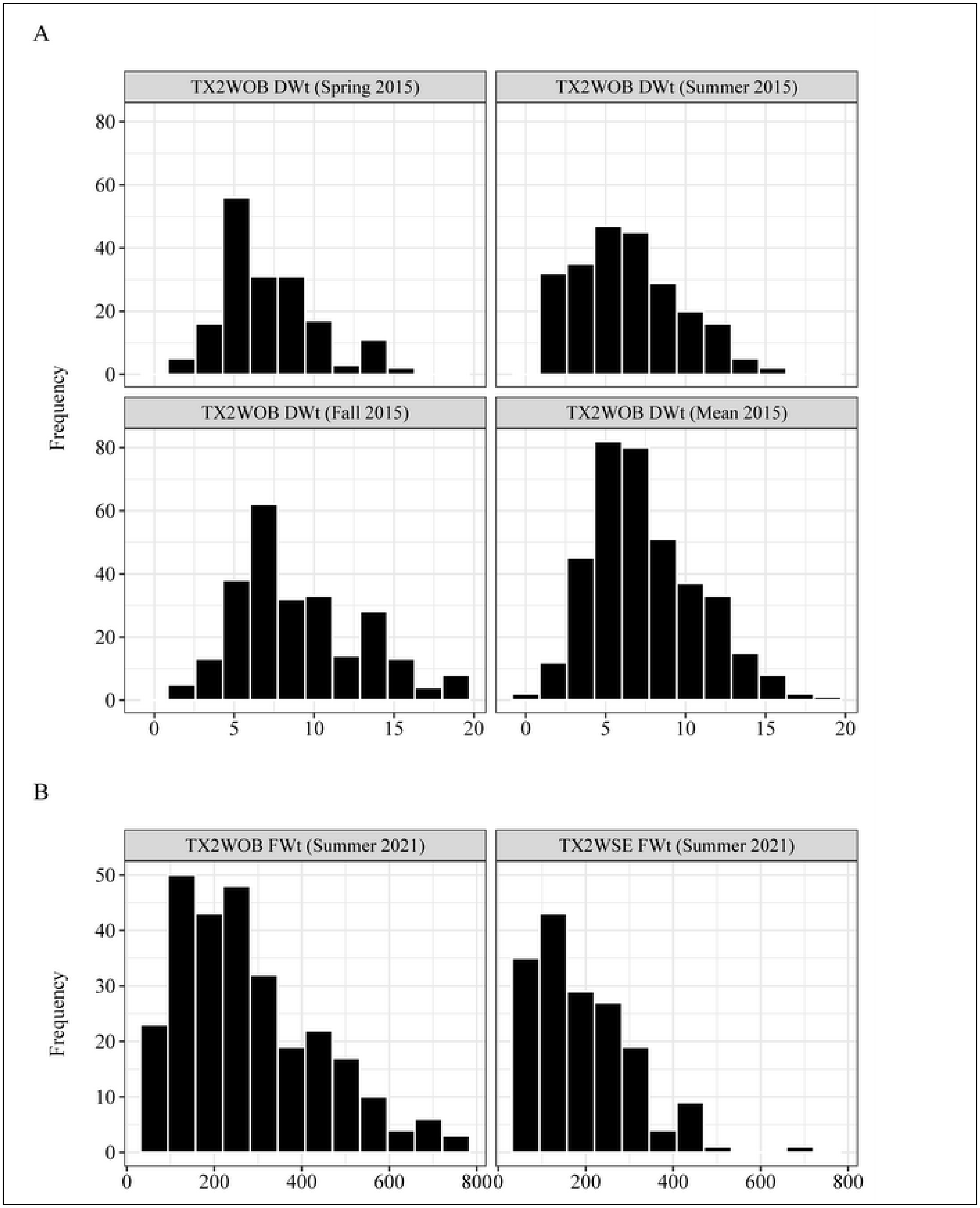

Supplementary Figure 5. Histograms for flower dry weight phenotyped in spring, summer, and fall in 2015 for the TX2WOB diploid rose population (A) and fresh weight in summer 2021 for TX2WOB and TX2WSE (B).
