# Supplementary material for: Pedigree-based QTL analysis of flower size traits in two multi-parental diploid rose populations
Source: Front Plant Sci. 2023 Aug 15;14:1226713. doi: 10.3389/fpls.2023.1226713 (PMC10464838; doi:10.3389/fpls.2023.1226713)
Supplement: Supplementary file 6 [file Image_6.pdf]

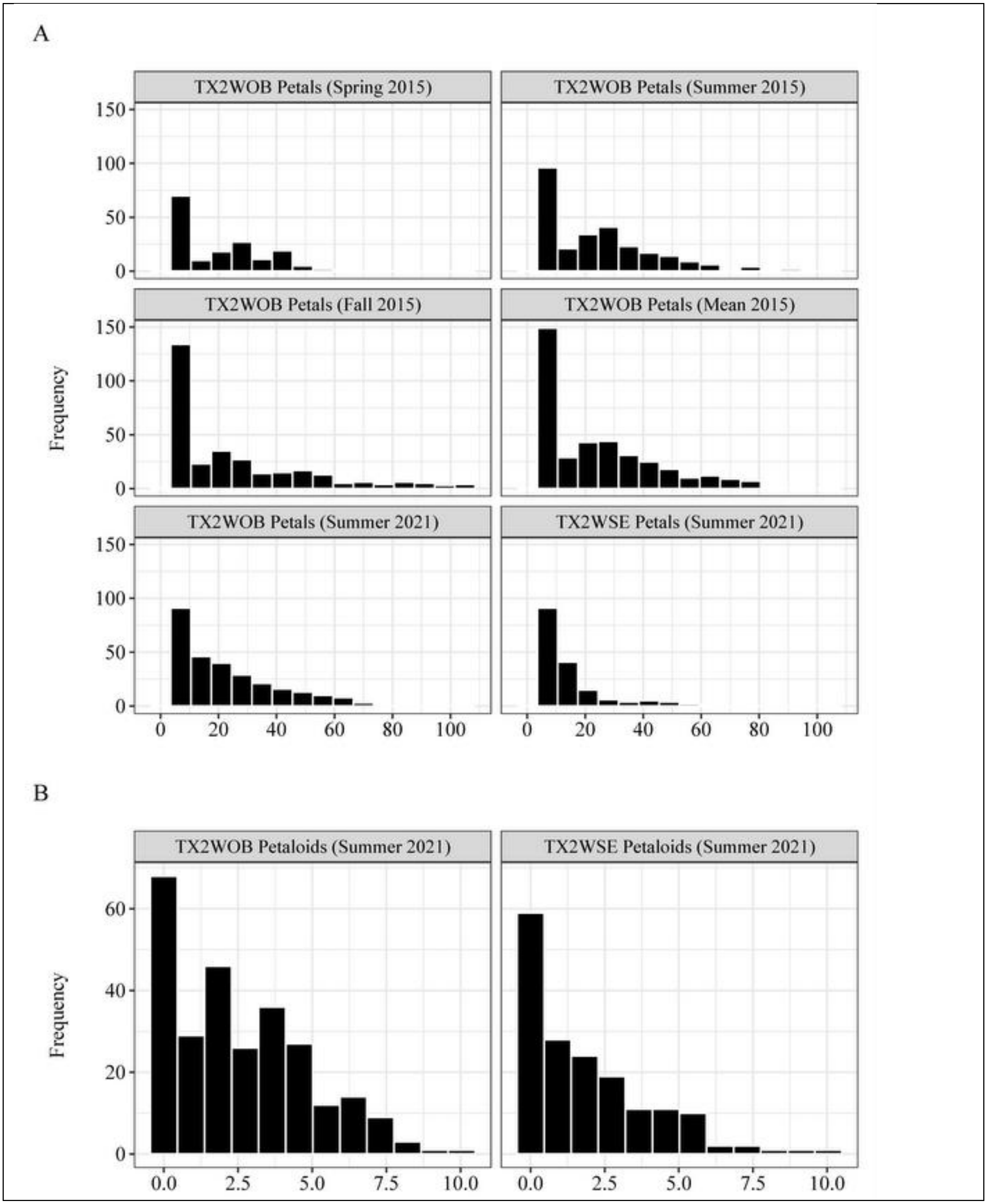

Supplementary Figure 6. Histograms for number of petals phenotyped in spring, summer, and fall in 2015 for the TX2WOB diploid rose population and in summer 2021 for TX2WOB and TX2WSE (A), and number of petaloid in summer 2021 for TX2WOB and TX2WSE (B).
