# Supplementary material for: Pedigree-based QTL analysis of flower size traits in two multi-parental diploid rose populations
Source: Front Plant Sci. 2023 Aug 15;14:1226713. doi: 10.3389/fpls.2023.1226713 (PMC10464838; doi:10.3389/fpls.2023.1226713)
Supplement: Supplementary file 7 [file Image_7.pdf]

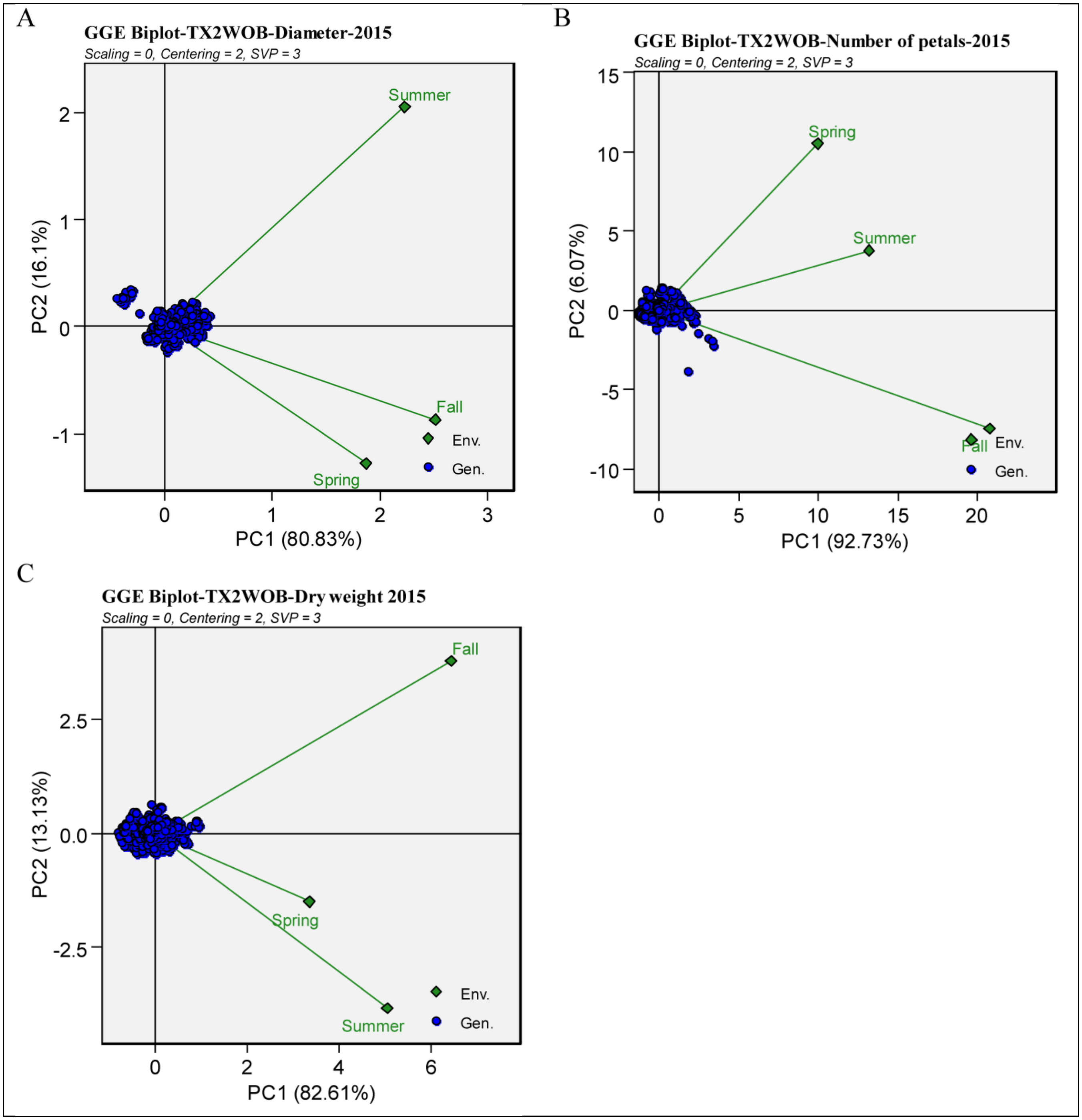

Supplementary Figure 7. Genotype plus genotype × environment (GGE) biplot representing the discrimination and representativeness of environments (seasons) for diameter (A), number of petals (B), and dry weight (C) phenotyped in Texas College Station (CS) in spring, summer, and fall 2015 for TX2WOB population.
