# Supplementary material for: Pedigree-based QTL analysis of flower size traits in two multi-parental diploid rose populations
Source: Front Plant Sci. 2023 Aug 15;14:1226713. doi: 10.3389/fpls.2023.1226713 (PMC10464838; doi:10.3389/fpls.2023.1226713)
Supplement: Supplementary file 8 [file Image_8.pdf]

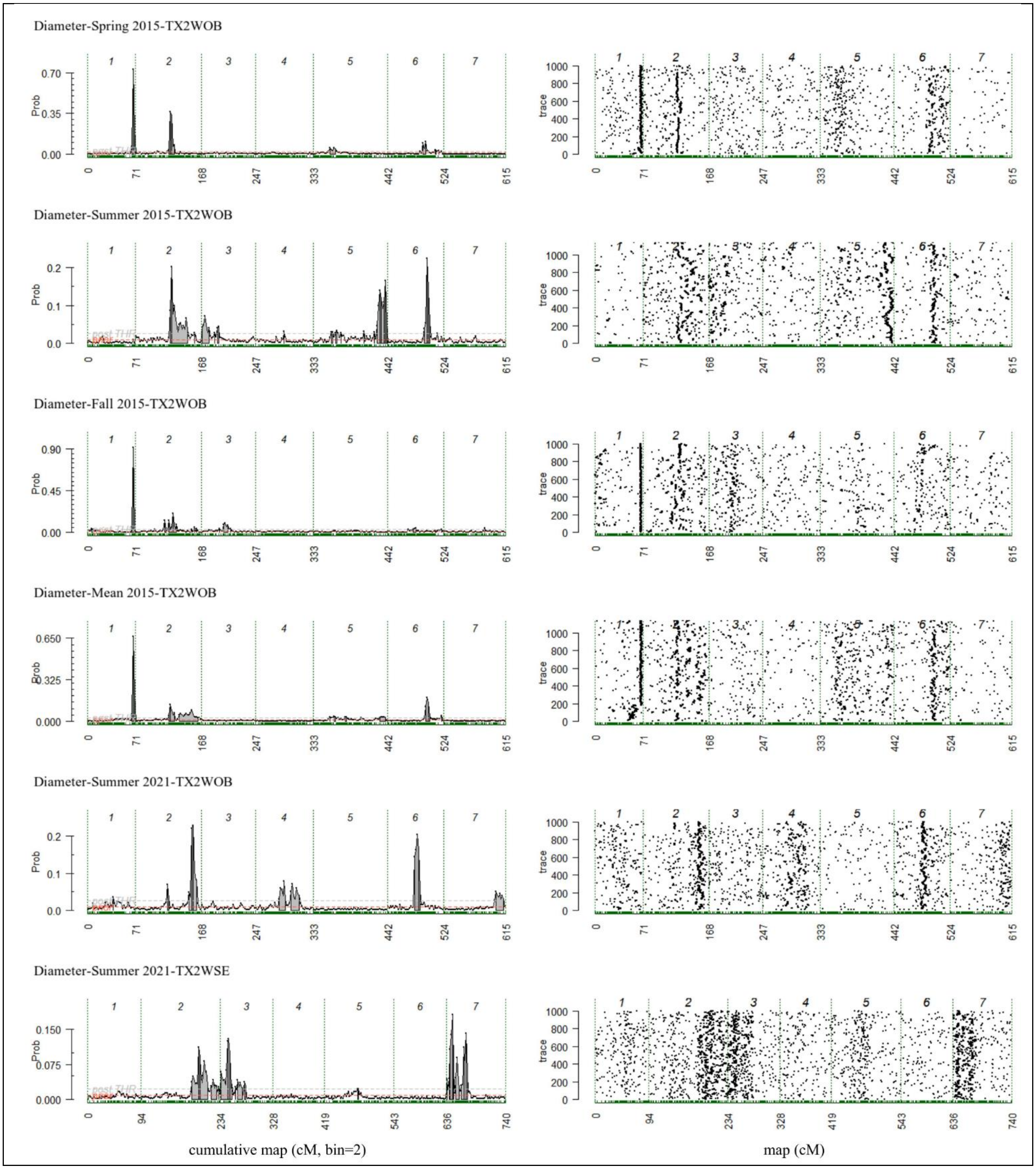

Supplementary Figure 8. Posterior positions (left) and trace samples QTL positions (right) based on an additive model performed using Visual FlexQTL software for diameter phenotyped in Texas in spring, summer, fall, and the mean in 2015 for TX2WOB diploid rose population in College Station, and in summer 2021 for TX2WOB and TX2WSE in Somerville.
