# Supplementary material for: Pedigree-based QTL analysis of flower size traits in two multi-parental diploid rose populations
Source: Front Plant Sci. 2023 Aug 15;14:1226713. doi: 10.3389/fpls.2023.1226713 (PMC10464838; doi:10.3389/fpls.2023.1226713)
Supplement: Supplementary file 11 [file Image_11.pdf]

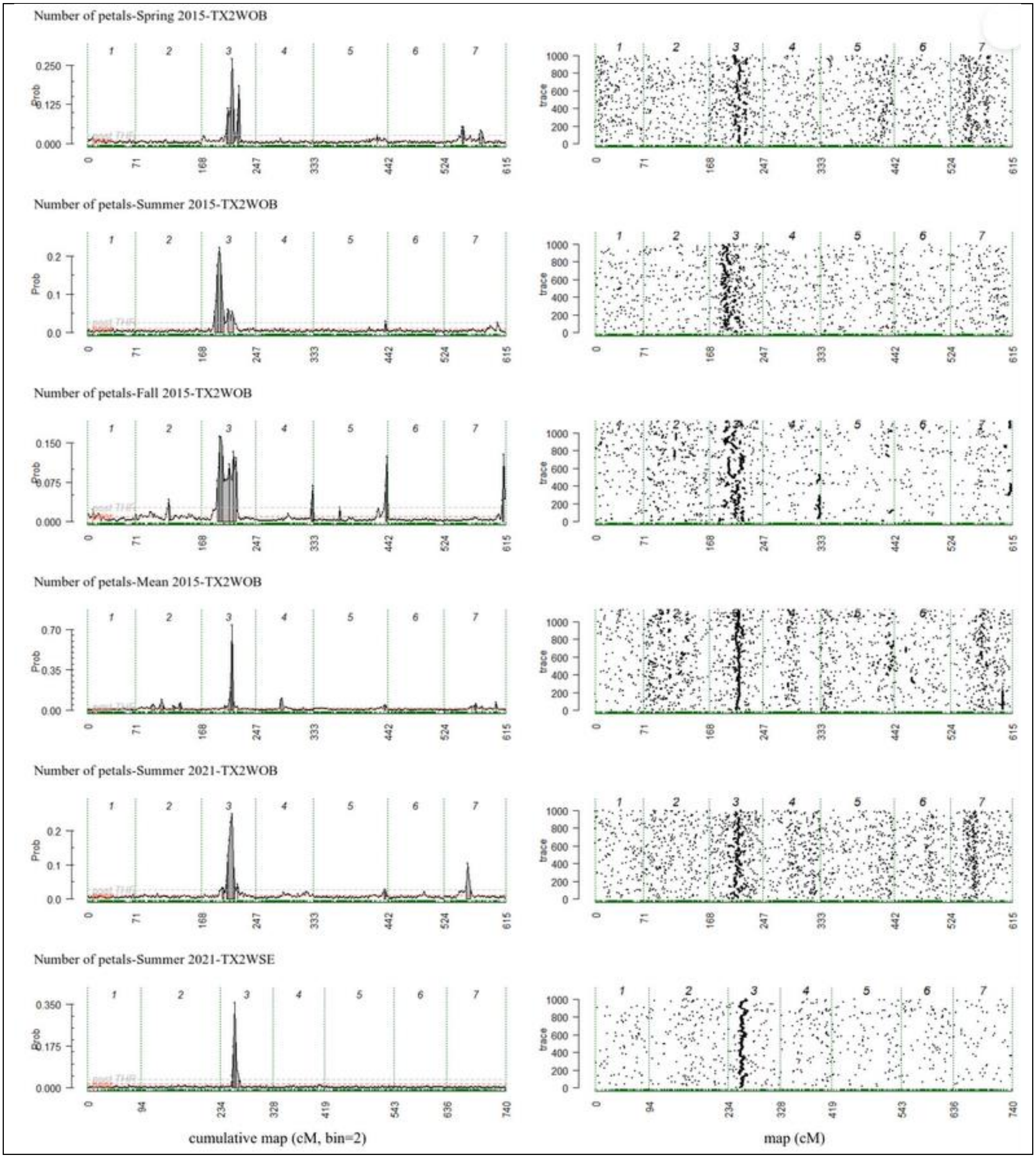

Supplementary Figure 11. Posterior positions (left) and trace samples QTL positions (right) based on an additive model performed using Visual FlexQTL software for number of petals phenotyped in Texas in spring, summer, fall, and the mean in 2015 for TX2WOB diploid rose population in College Station, and in summer 2021 for TX2WOB and TX2WSE in Somerville. (QTL analysis with co-factor).
