# Supplementary material for: Pedigree-based QTL analysis of flower size traits in two multi-parental diploid rose populations
Source: Front Plant Sci. 2023 Aug 15;14:1226713. doi: 10.3389/fpls.2023.1226713 (PMC10464838; doi:10.3389/fpls.2023.1226713)
Supplement: Supplementary file 12 [file Image_12.pdf]

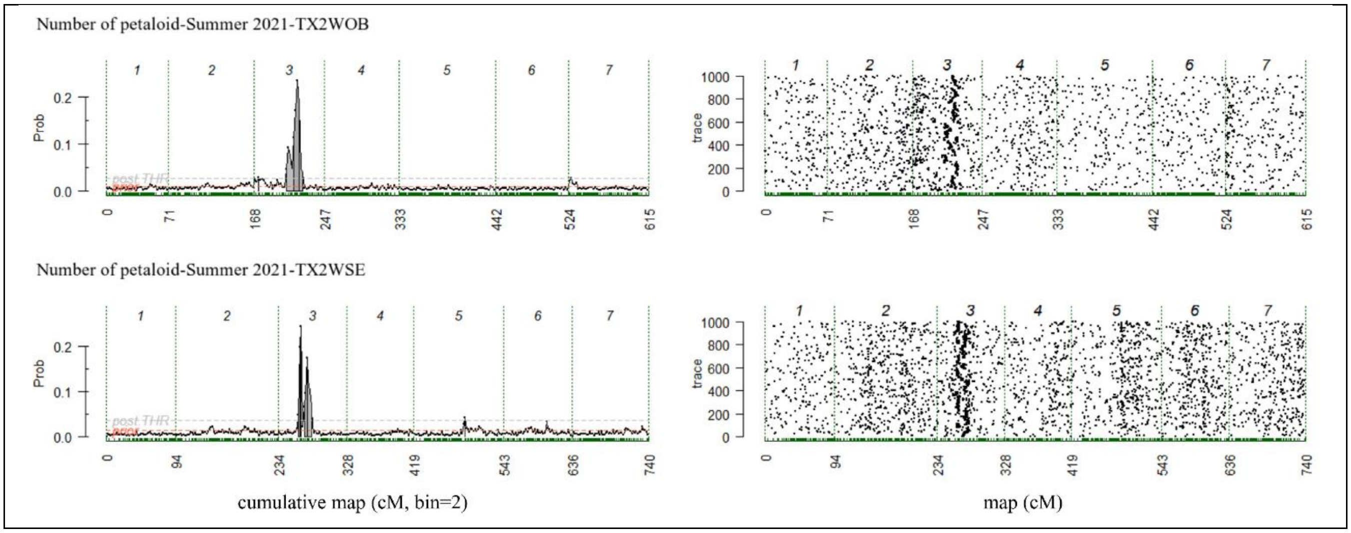

Supplementary Figure 12. Posterior positions (left) and trace samples QTL positions (right) based on an additive model performed using Visual FlexQTL software for number of petaloid phenotyped in Somerville, Texas in summer 2021 for TX2WOB and TX2WSE diploid rose populations.
