# Supplementary material for: Pedigree-based QTL analysis of flower size traits in two multi-parental diploid rose populations
Source: Front Plant Sci. 2023 Aug 15;14:1226713. doi: 10.3389/fpls.2023.1226713 (PMC10464838; doi:10.3389/fpls.2023.1226713)
Supplement: Supplementary file 13 [file Image_13.pdf]

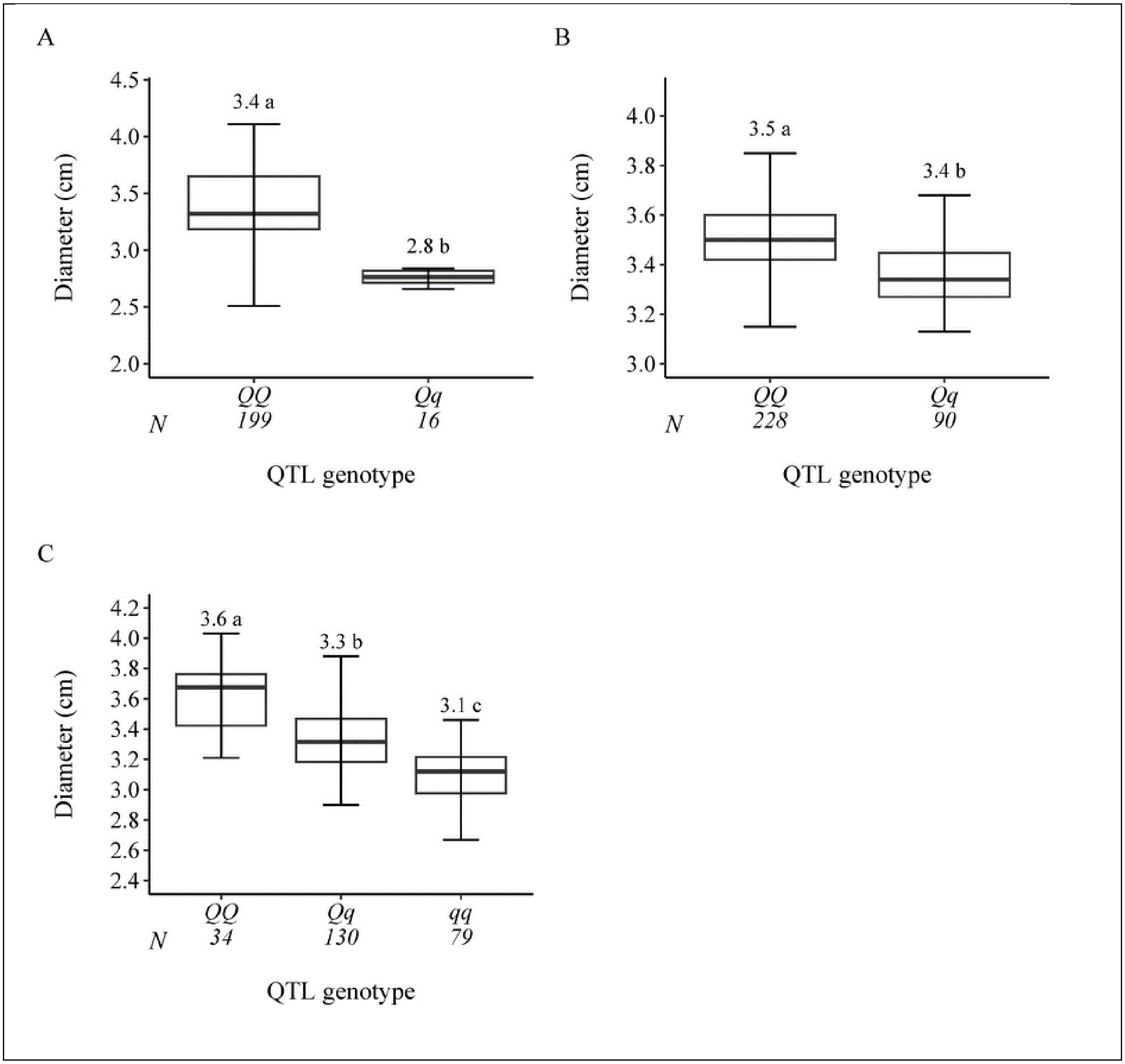

Supplementary Figure 13. Probable QTL genotype at the signal peak from all progenies for diameter QTLs of diploid rose populations *q*Diam.TX2WOB-LG1 (A) and *q*Diam.TX2WOB-LG2.2 (B) for TX2WOB, and *q*Diam.TX2WSE-LG2 (C) for TX2WSE.

Means not connected by the same letter are significantly different ($P<0.05$) within each linkage group.
