# Supplementary material for: Pedigree-based QTL analysis of flower size traits in two multi-parental diploid rose populations
Source: Front Plant Sci. 2023 Aug 15;14:1226713. doi: 10.3389/fpls.2023.1226713 (PMC10464838; doi:10.3389/fpls.2023.1226713)
Supplement: Supplementary file 14 [file Image_14.pdf]

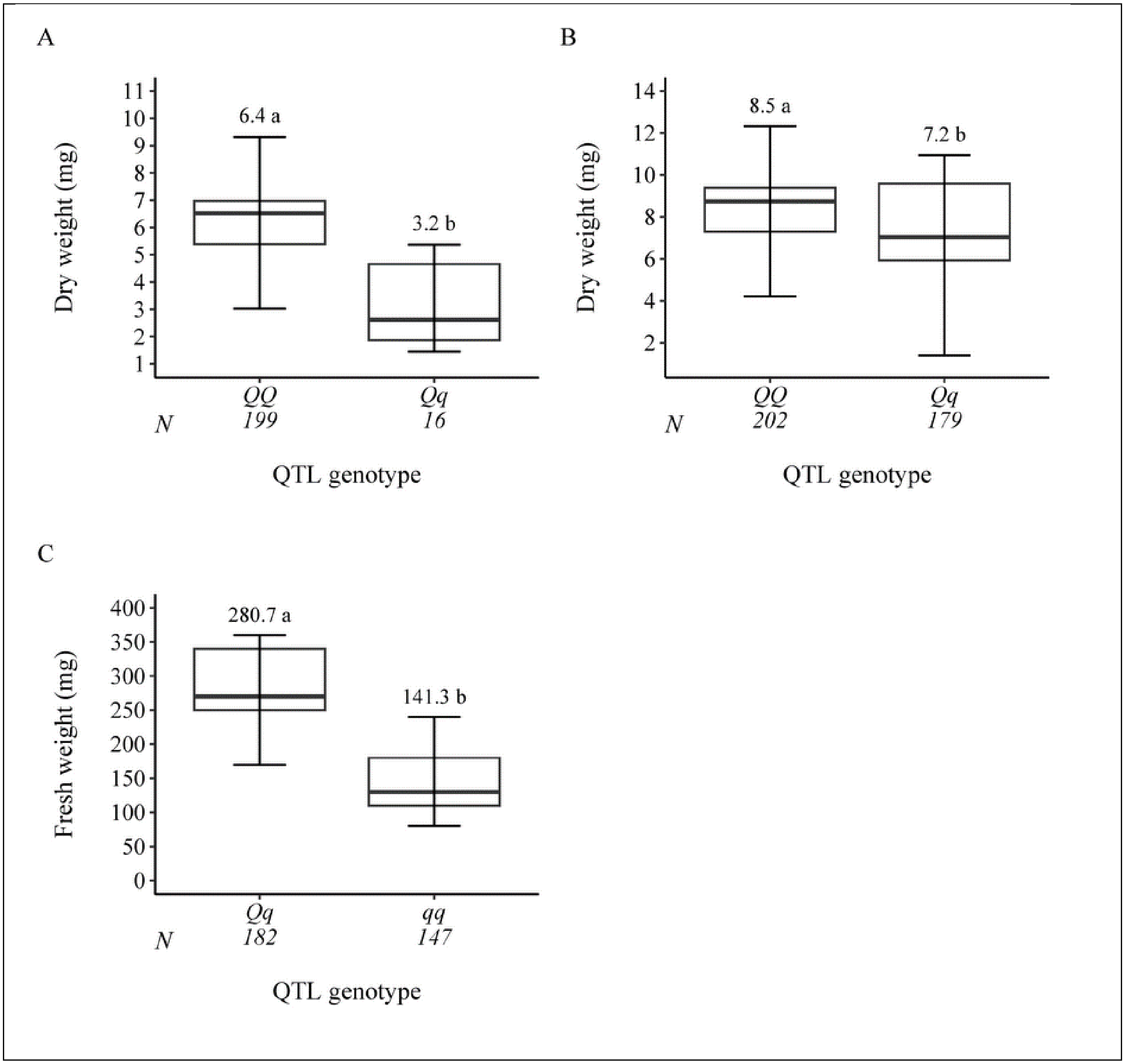

Supplementary Figure 14. Probable QTL genotype at the signal peak from all progenies for flower dry weight and fresh weight QTLs of diploid rose populations *q*DWT.TX2WOB-LG1(A) and *q*DWT.TX2WOB-LG3 (B) for TX2WOB and *q*FWT.TX2WSE-LG3 (C) for TX2WSE.

Means not connected by the same letter are significantly different ($P<0.05$) within each linkage group.
