# Supplementary material for: Pedigree-based QTL analysis of flower size traits in two multi-parental diploid rose populations
Source: Front Plant Sci. 2023 Aug 15;14:1226713. doi: 10.3389/fpls.2023.1226713 (PMC10464838; doi:10.3389/fpls.2023.1226713)
Supplement: Supplementary file 17 [file Image_17.pdf]

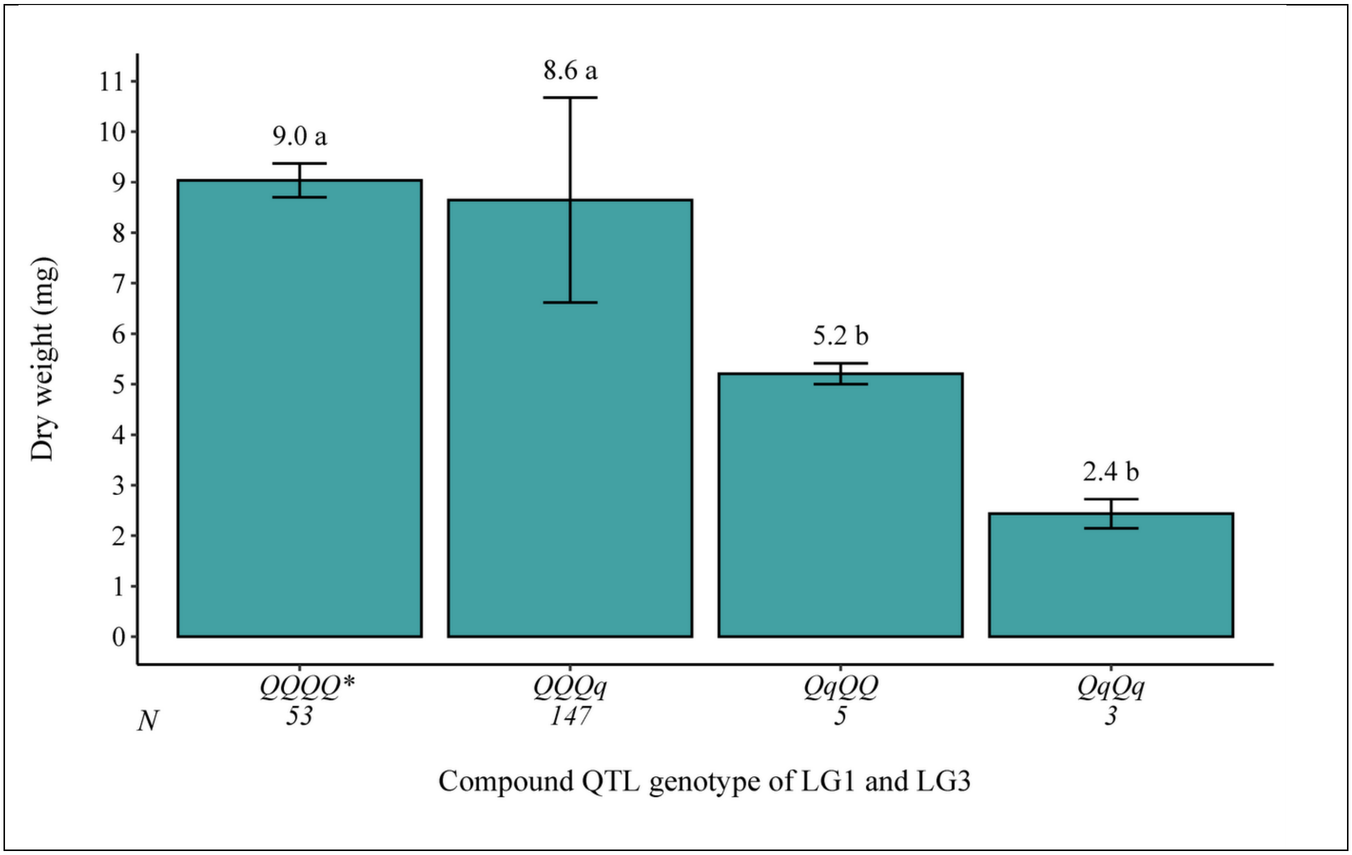

Supplementary Figure 17. Analysis of the compound QTL genotypes from *q*DWT.TX2WOB-LG1 and *q*DWT.TX2WOB-LG3 on dry weight from all progenies in TX2WOB population.
Means not connected by the same letter are significantly different ($P<0.05$).

* The 1st and 2nd pairs of QTL alleles are for the LG1 and LG3 QTLs, respectively.

N = Sample size
