# Supplementary material for: Pedigree-based QTL analysis of flower size traits in two multi-parental diploid rose populations
Source: Front Plant Sci. 2023 Aug 15;14:1226713. doi: 10.3389/fpls.2023.1226713 (PMC10464838; doi:10.3389/fpls.2023.1226713)
Supplement: Supplementary file 18 [file Image_18.pdf]

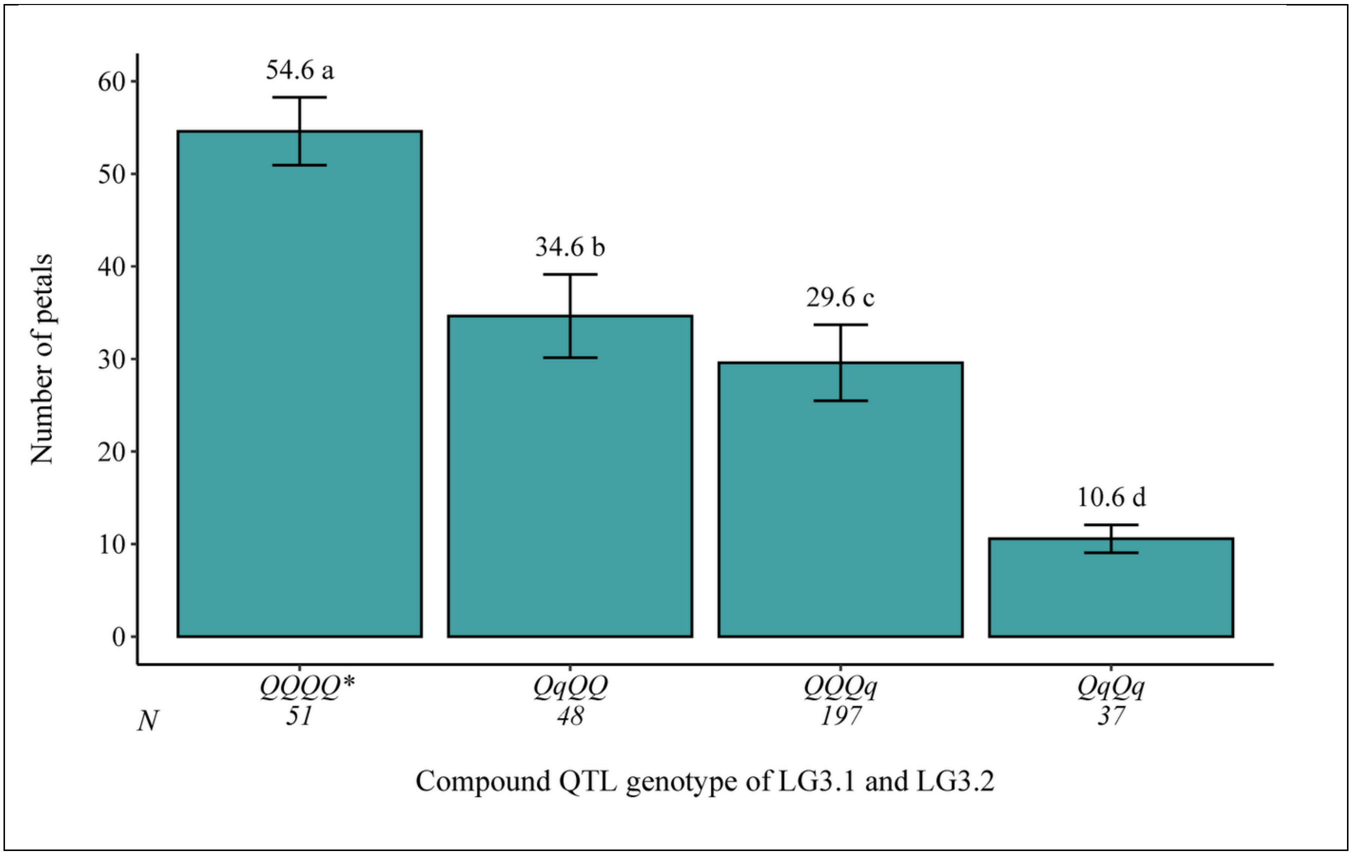


**Supplementary Figure 18.** Analysis of the compound QTL genotypes from *q*NP.TX2WOB-LG3.1 and *q*NP.TX2WOB-LG3.2 on number of petals from all progenies in TX2WOB population.
Means not connected by the same letter are significantly different ($P<0.05$).

* The 1st and 2nd pairs of QTL alleles are for the LG3.1 and LG3.2 QTLs, respectively.

N = Sample size
