# Supplementary material for: Pedigree-based QTL analysis of flower size traits in two multi-parental diploid rose populations
Source: Front Plant Sci. 2023 Aug 15;14:1226713. doi: 10.3389/fpls.2023.1226713 (PMC10464838; doi:10.3389/fpls.2023.1226713)
Supplement: Supplementary file 19 [file Table_1.docx]

| **Supplementary Table 1**. Parentage and number of progenies for diploid rose multi-parental populations used for QTL analysis in College Station and Somerville, Texas in 2015 and 2021 for TX2WOB (A), and in 2021 Somerville, Texas for TX2WSE (B). | | | | | | | | | |
| --- | --- | --- | --- | --- | --- | --- | --- | --- | --- |
| A | | | | |  | B | | | |
| Family | Female Parent | Male Parent | 2015 | 2021 |  | Family | Female Parent | Male Parent | 2021 |
| J14-3×LC | J06-20-14-3 | Little Chief | 64 | 31 |  | J14-3×PH | J06-20-14-3 | Papa Hemeray | 117 |
| J14-3×RF | J06-28-4-6 | Red Fairy | - | 34 |  | M4-4×SEimp | M4-4 | Srdce Europy | 32 |
| J14-3×SC | J06-20-14-3 | Sweet Chariot | - | 41 |  | PH×SEB-ARE | Papa Hemeray | *R. palustris* f. *plena* EB-ARE | 10 |
| J14-3×VS | J06-20-14-3 | Vineyard Song | 84 | 49 |  | SET-ARE×OL | *R. setigera*-ARE | Ole | 20 |
| M4-4×SC | M4-4 | Sweet Chariot | - | 5 |  | T7-20×SEimp | TAMU7-20 | Srdce Europy | 93 |
| OB×J3-6 | Old Blush | J06-30-3-6 | 95 | 44 |  | T7-30×SEimp | TAMU7-30 | Srdce Europy | 81 |
| OB×M4-4 | Old Blush | M4-4 | - | 8 |  |  |  |  |  |
| OB×RF | Old Blush | Red Fairy | 79 | 43 |  |  |  |  |  |
| SC×J14-3 | Sweet Chariot | J06-20-14-3 | - | 21 |  |  |  |  |  |
| SC×M4-4 | Sweet Chariot | M4-4 | - | 24 |  |  |  |  |  |
| J4-6×RF | J06-20-14-3 | Red Fairy | 65 | - |  |  |  |  |  |
| Total |  |  | 387 | 300 |  |  |  |  | 353 |
