# Supplementary material for: Pedigree-based QTL analysis of flower size traits in two multi-parental diploid rose populations
Source: Front Plant Sci. 2023 Aug 15;14:1226713. doi: 10.3389/fpls.2023.1226713 (PMC10464838; doi:10.3389/fpls.2023.1226713)
Supplement: Supplementary file 20 [file Table_2.docx]

| **Supplementary Table 2**. Monthly maximum (Max), minimum (Min), and average temperature in College Station in 2015 and Somerville in 2021 in Texas. | | | | | | |
| --- | --- | --- | --- | --- | --- | --- |
|  | 2015 | | | 2021 | | |
| Month/Season | Max | Min | Avg | Max | Min | Avg |
| Jan | 13.5 | 3.9 | 8.4 | 17.2 | 6.0 | 11.4 |
| Feb | 15.8 | 5.2 | 10.1 | 14.2 | 3.9 | 8.9 |
| Mar | 20.1 | 10.6 | 15.2 | 23.4 | 11.8 | 17.5 |
| Apr | 26.1 | 16.9 | 21.2 | 24.9 | 14.9 | 19.7 |
| May | 28.4 | 19.9 | 23.8 | 28.4 | 20.0 | 23.8 |
| Jun | 32.0 | 23.1 | 27.2 | 32.6 | 23.9 | 27.9 |
| Jul | 34.6 | 24.3 | 29.2 | 32.9 | 24.0 | 27.9 |
| Aug | 35.6 | 23.9 | 29.3 | 34.4 | 24.9 | 29.1 |
| Sep | 33.0 | 22.1 | 26.8 | 33.4 | 21.6 | 27.0 |
| Oct | 29.2 | 17.4 | 23.0 | 29.3 | 18.2 | 23.3 |
| Nov | 21.3 | 11.9 | 16.5 | 22.3 | 10.3 | 16.1 |
| Dec | 19.8 | 8.9 | 14.1 | 24.1 | 14.5 | 18.8 |
| Spring | 24.8 | 15.8 | 20.0 | 25.6 | 15.6 | 20.3 |
| Summer | 34.1 | 23.8 | 28.6 | 33.3 | 24.3 | 28.3 |
| Fall | 27.8 | 17.1 | 22.1 | 28.3 | 16.7 | 22.2 |
