# Supplementary material for: Pedigree-based QTL analysis of flower size traits in two multi-parental diploid rose populations
Source: Front Plant Sci. 2023 Aug 15;14:1226713. doi: 10.3389/fpls.2023.1226713 (PMC10464838; doi:10.3389/fpls.2023.1226713)
Supplement: Supplementary file 21 [file Table_3.docx]

| **Supplementary Table 3**. Statistical summary of the five individual diploid rose maps and the integrated consensus map (TX2WOB ICM) by linkage group (LG). | | | | | | | |
| --- | --- | --- | --- | --- | --- | --- | --- |
|  | | | | | | Unique positions | |
| Family | LG | SNP | Length (cM) | Max gap (cM) | Density | SNP | Density |
| J14-3×LC | 1 | 532 | 70.6 | 7.1 | 7.5 | 177 | 2.5 |
| N=73^*^ | 2 | 405 | 96.3 | 5.4 | 4.2 | 151 | 1.6 |
|  | 3 | 342 | 75.8 | 10.3 | 4.5 | 125 | 1.6 |
|  | 4 | 247 | 73.8 | 8.2 | 3.3 | 131 | 1.8 |
|  | 5 | 189 | 107.1 | 11.3 | 1.8 | 110 | 1.0 |
|  | 6 | 386 | 82.3 | 11.2 | 4.7 | 178 | 2.2 |
|  | 7 | 256 | 90.7 | 5.1 | 2.8 | 101 | 1.1 |
| Total | | 2,357 | 596.6 | 11.3 | 4.1 | 973 | 1.7 |
| J14-3×VS | 1 | 406 | 84.6 | 14.0 | 4.8 | 147 | 1.7 |
| N=89 | 2 | 362 | 97.1 | 7.9 | 3.7 | 144 | 1.5 |
|  | 3 | 218 | 79.6 | 14.6 | 2.7 | 81 | 1.0 |
|  | 4 | 329 | 71.9 | 8.5 | 4.6 | 153 | 2.1 |
|  | 5 | 192 | 99.5 | 11.1 | 1.9 | 118 | 1.2 |
|  | 6 | 279 | 71.1 | 4.2 | 3.9 | 143 | 2.0 |
|  | 7 | 221 | 79.1 | 6.5 | 2.8 | 101 | 1.3 |
| Total | | 2,007 | 582.9 | 14.6 | 3.5 | 887 | 1.5 |
| J4-6×RF | 1 | 295 | 70.6 | 7.7 | 4.2 | 120 | 1.7 |
| N=68 | 2 | 321 | 95.6 | 8.4 | 3.4 | 134 | 1.4 |
|  | 3 | 196 | 74.4 | 13.8 | 2.6 | 80 | 1.1 |
|  | 4 | 352 | 85.9 | 8.7 | 4.1 | 149 | 1.7 |
|  | 5 | 200 | 108.9 | 5.3 | 1.8 | 100 | 0.9 |
|  | 6 | 243 | 69.5 | 3.3 | 3.5 | 131 | 1.9 |
|  | 7 | 196 | 75.8 | 6.1 | 2.6 | 98 | 1.3 |
| Total | | 1,803 | 580.7 | 13.8 | 3.2 | 812 | 1.4 |
| OB×J3-6 | 1 | 515 | 87.7 | 17.1 | 5.9 | 183 | 2.1 |
| N=97 | 2 | 242 | 83.4 | 9.4 | 2.9 | 94 | 1.1 |
|  | 3 | 147 | 64.1 | 6.9 | 2.3 | 65 | 1.0 |
|  | 4 | 214 | 71.4 | 9.8 | 3.0 | 108 | 1.5 |
|  | 5 | 378 | 97.9 | 5.5 | 3.9 | 203 | 2.1 |
|  | 6 | 311 | 75.6 | 6.7 | 4.1 | 165 | 2.2 |
|  | 7 | 104 | 36.0 | 3.5 | 2.9 | 52 | 1.4 |
| Total | | 1,911 | 516.1 | 17.1 | 3.6 | 870 | 1.6 |
| OB×RF | 1 | 222 | 70.6 | 8.6 | 3.1 | 69 | 1.0 |
| N=88 | 2 | 401 | 94.6 | 11.9 | 4.2 | 154 | 1.6 |
|  | 3 | 114 | 74.6 | 13.2 | 1.5 | 56 | 0.8 |
|  | 4 | 316 | 85.9 | 4.3 | 3.7 | 203 | 2.4 |
|  | 5 | 443 | 97.9 | 5.5 | 4.5 | 225 | 2.3 |
|  | 6 | 56 | 103.2 | 28.5 | 0.5 | 52 | 0.5 |
|  | 7 | 137 | 89.4 | 30.8 | 1.5 | 74 | 0.8 |
| Total | | 1,689 | 616.2 | 30.8 | 2.7 | 833 | 1.3 |
| Consensus | 1 | 757 | 87.7 | 14.0 | 8.6 | 252 | 2.9 |
| N=415 | 2 | 779 | 97.1 | 3.8 | 8.0 | 308 | 3.2 |
|  | 3 | 518 | 79.6 | 3.5 | 6.5 | 198 | 2.5 |
|  | 4 | 662 | 85.9 | 4.0 | 7.7 | 319 | 3.7 |
|  | 5 | 625 | 108.9 | 4.8 | 5.7 | 318 | 2.9 |
|  | 6 | 619 | 103.2 | 9.4 | 6.0 | 288 | 2.8 |
|  | 7 | 507 | 90.7 | 4.9 | 5.6 | 213 | 2.3 |
| Total | | 4,467 | 653.1 | 14.0 | 6.9 | 1,896 | 2.9 |
| * Number of individuals  LC = ‘Little Chief’, VS=’Vineyard Song’, RF=‘Red Fairy’, OB=’Old Blush’, and SC=’Sweet Chariot’ | | | | | | | |
