# Supplementary material for: Pedigree-based QTL analysis of flower size traits in two multi-parental diploid rose populations
Source: Front Plant Sci. 2023 Aug 15;14:1226713. doi: 10.3389/fpls.2023.1226713 (PMC10464838; doi:10.3389/fpls.2023.1226713)
Supplement: Supplementary file 22 [file Table_4.docx]

| **Supplementary Table 4**. Statistical summary of the three individual diploid rose maps and the integrated consensus map (TX2WSE ICM) by linkage group (LG). | | | | | | | |
| --- | --- | --- | --- | --- | --- | --- | --- |
|  | | | | | | Unique positions | |
| Family | LG | SNP | Length (cM) | Max gap (cM) | Density | SNP | Density |
| J14-3×PH | 1 | 949 | 95.7 | 3.2 | 9.9 | 876 | 9.2 |
| N=138^*^ | 2 | 1,179 | 146.1 | 2.6 | 8.1 | 1,076 | 7.4 |
|  | 3 | 1,093 | 94.1 | 4.2 | 11.6 | 976 | 10.4 |
|  | 4 | 1,017 | 93.6 | 1.8 | 10.9 | 919 | 9.8 |
|  | 5 | 1,474 | 130.6 | 2.9 | 11.3 | 1,356 | 10.4 |
|  | 6 | 1,713 | 89.0 | 3.2 | 19.2 | 1,358 | 15.3 |
|  | 7 | 871 | 110.4 | 1.9 | 7.9 | 801 | 7.3 |
| Total | | 8,296 | 759.5 | 4.2 | 10.9 | 7,362 | 9.7 |
| T7-20×SE | 1 | 390 | 75.8 | 1.6 | 5.1 | 359 | 4.7 |
| N=94 | 2 | 677 | 97.6 | 5.5 | 6.9 | 561 | 5.7 |
|  | 3 | 688 | 87.2 | 2.5 | 7.9 | 604 | 6.9 |
|  | 4 | 506 | 78.2 | 2.2 | 6.5 | 458 | 5.9 |
|  | 5 | 816 | 107.6 | 2.2 | 7.6 | 757 | 7.0 |
|  | 6 | 1,125 | 94.5 | 3.0 | 11.9 | 887 | 9.4 |
|  | 7 | 816 | 87.3 | 3.2 | 9.3 | 733 | 8.4 |
| Total | | 5,018 | 628.2 | 5.5 | 8.0 | 4,359 | 6.9 |
| T7-30×SE | 1 | 677 | 97.0 | 1.9 | 7.0 | 619 | 6.4 |
| N=82 | 2 | 1,149 | 146.5 | 5.7 | 7.8 | 999 | 6.8 |
|  | 3 | 730 | 95.5 | 3.1 | 7.6 | 660 | 6.9 |
|  | 4 | 565 | 88.5 | 5.9 | 6.4 | 510 | 5.8 |
|  | 5 | 748 | 104.7 | 2.8 | 7.1 | 694 | 6.6 |
|  | 6 | 1,141 | 103.8 | 2.3 | 11.0 | 953 | 9.2 |
|  | 7 | 954 | 119.9 | 2.8 | 8.0 | 877 | 7.3 |
| Total | | 5,964 | 755.8 | 5.9 | 7.9 | 5,312 | 7.0 |
| Consensus | 1 | 346 | 97.7 | 3.4 | 3.5 | 168 | 1.7 |
| N=314 | 2 | 467 | 146.1 | 4.5 | 3.2 | 197 | 1.3 |
|  | 3 | 345 | 93.9 | 3.4 | 3.7 | 141 | 1.5 |
|  | 4 | 321 | 93.6 | 4.5 | 3.4 | 139 | 1.5 |
|  | 5 | 417 | 123.6 | 5.9 | 3.4 | 194 | 1.6 |
|  | 6 | 389 | 92.8 | 4.2 | 4.2 | 178 | 1.9 |
|  | 7 | 392 | 110.6 | 6.8 | 3.5 | 145 | 1.3 |
| Total | | 2,677 | 758.2 | 6.8 | 3.5 | 1,162 | 1.5 |
| * Number of individuals | | | | | | | |
