# Supplementary material for: Pedigree-based QTL analysis of flower size traits in two multi-parental diploid rose populations
Source: Front Plant Sci. 2023 Aug 15;14:1226713. doi: 10.3389/fpls.2023.1226713 (PMC10464838; doi:10.3389/fpls.2023.1226713)
Supplement: Supplementary file 23 [file Table_5.docx]

| **Supplementary Table 5**. Descriptive statistics of the diameter (Diam), dry weight (DWT), fresh weight (FWT), number of petals (NP), and number of petaloid (PD) phenotyped in different seasons in Texas on diploid rose multi-parental populations, TX2WOB, in 2015 in College Station (CS) and 2021 in Somerville (SV) and TX2WSE in 2021. | | | | | | | | |
| --- | --- | --- | --- | --- | --- | --- | --- | --- |
| Trait | Population | Year | Season | N | Mean | Var | Min | Max |
| Diam | TX2WOB | 2015 | Spring | 179 | 3.8 | 0.39 | 1.5 | 5.6 |
|  | TX2WOB | 2015 | Summer | 270 | 3.6 | 0.41 | 2.0 | 5.4 |
|  | TX2WOB | 2015 | Fall | 262 | 4.1 | 0.58 | 2.3 | 6.1 |
|  | TX2WOB | 2015 | Mean | 309 | 3.7 | 0.46 | 2.0 | 7.2 |
|  | TX2WOB | 2021 | Summer | 277 | 3.5 | 0.31 | 2.0 | 5.0 |
|  | TX2WSE | 2021 | Summer | 169 | 3.3 | 0.32 | 2.2 | 4.8 |
| DWT | TX2WOB | 2015 | Spring | 172 | 7.2 | 7.92 | 1.0 | 15.0 |
|  | TX2WOB | 2015 | Summer | 231 | 6.4 | 10.21 | 1.1 | 14.8 |
|  | TX2WOB | 2015 | Fall | 250 | 9.0 | 15.39 | 2.0 | 19.5 |
|  | TX2WOB | 2015 | Mean | 368 | 7.5 | 10.91 | 0.6 | 18.1 |
| FWT | TX2WOB | 2021 | Summer | 277 | 280.0 | 26,000.00 | 40.0 | 730.0 |
|  | TX2WSE | 2021 | Summer | 168 | 190.0 | 13,000.00 | 40.0 | 710.0 |
| NP | TX2WOB | 2015 | Spring | 163 | 20.3 | 232.27 | 5.0 | 63.7 |
|  | TX2WOB | 2015 | Summer | 269 | 23.8 | 355.43 | 5.0 | 92.1 |
|  | TX2WOB | 2015 | Fall | 316 | 27.3 | 825.27 | 1.0 | 183.4 |
|  | TX2WOB | 2015 | Mean | 377 | 23.5 | 382.50 | 4.0 | 78.1 |
|  | TX2WOB | 2021 | Summer | 277 | 22.4 | 278.65 | 4.8 | 69.1 |
|  | TX2WSE | 2021 | Summer | 169 | 14.1 | 141.80 | 4.8 | 66.0 |
| PD | TX2WOB | 2021 | Summer | 272 | 2.6 | 5.01 | 0.0 | 9.8 |
|  | TX2WSE | 2021 | Summer | 169 | 1.9 | 4.40 | 0.0 | 10.0 |
