# Supplementary material for: Pedigree-based QTL analysis of flower size traits in two multi-parental diploid rose populations
Source: Front Plant Sci. 2023 Aug 15;14:1226713. doi: 10.3389/fpls.2023.1226713 (PMC10464838; doi:10.3389/fpls.2023.1226713)
Supplement: Supplementary file 24 [file Table_6.docx]

| **Supplementary Table 6**. Genetic variance ${(\sigma}_{g}^{2})$, genotype by environment variance ${(\sigma}_{g\times e}^{2})$, the genotype by environment variance relative to the genetic variance $({\sigma_{g\times e}^{2}}/{\sigma_{g}^{2})}$, and broad-sense heritability (*H^2^*) for diameter (Diam), dry weight (DWT), and number of petals (NP) in five diploid rose populations (TX2WOB) phenotyped in College Station, Texas over three seasons (spring, summer, and fall) in 2015. | | | | |
| --- | --- | --- | --- | --- |
| Trait | $\sigma_{g}^{2}$ | $\sigma_{g\times e}^{2}$ | ${\sigma_{g\times e}^{2}}/{\sigma_{g}^{2}}$ | *H^2^* |
| Diam | 0.27 | 0.27 | 1.00 | 0.75 |
| DWT | 8.50 | 5.53 | 0.65 | 0.82 |
| NP | 396.84 | 170.17 | 0.43 | 0.87 |
