# Supplementary material for: Pedigree-based QTL analysis of flower size traits in two multi-parental diploid rose populations
Source: Front Plant Sci. 2023 Aug 15;14:1226713. doi: 10.3389/fpls.2023.1226713 (PMC10464838; doi:10.3389/fpls.2023.1226713)
Supplement: Supplementary file 25 [file Table_7.docx]

| **Supplementary Table 7**. Correlation coefficient (r) between environments for the diameter (Diam), dry weight (DWT), and the number of petals (NP) phenotyped in Texas on five and ten diploid rose populations (TX2WOB) in 2015 in College Station and in Somerville in 2021, respectively. | | | | |
| --- | --- | --- | --- | --- |
| Diam | Summer 2015 | Fall 2015 | Mean 2015 | Summer 2021 |
| Spring 2015 | 0.56** | 0.87** | 0.79** | 0.52** |
| Summer 2015 |  | 0.72** | 0.87** | 0.66** |
| Fall 2015 |  |  | 0.88** | 0.68** |
| Mean 2015 |  |  |  | 0.69** |
|  |  |  |  |  |
| NP | Summer 2015 | Fall 2015 | Mean 2015 | Summer 2021 |
| Spring 2015 | 0.87** | 0.74** | 0.91** | 0.75** |
| Summer 2015 |  | 0.91** | 0.92** | 0.93** |
| Fall 2015 |  |  | 0.85** | 0.88** |
| Mean 2015 |  |  |  | 0.83** |
|  |  |  |  |  |
| DWT | Summer 2015 | Fall 2015 | Mean 2015 |  |
| Spring 2015 | 0.80** | 0.72** | 0.86** |  |
| Summer 2015 |  | 0.70** | 0.88** |  |
| Fall 2015 |  |  | 0.83** |  |
