# Supplementary material for: Pedigree-based QTL analysis of flower size traits in two multi-parental diploid rose populations
Source: Front Plant Sci. 2023 Aug 15;14:1226713. doi: 10.3389/fpls.2023.1226713 (PMC10464838; doi:10.3389/fpls.2023.1226713)
Supplement: Supplementary file 26 [file Table_8.docx]

| **Supplementary Table 8**. Correlation coefficient (r) between traits for the diameter (Diam), dry weight (DWT), fresh weight (FWT), the number of petals (NP), and the number of petaloid (PD) phenotyped in Texas on five diploid rose populations (TX2WOB) in 2015 in College Station and on ten population of TX2WOB and six populations of TX2WSE in Somerville in 2021. | | | | | | |
| --- | --- | --- | --- | --- | --- | --- |
| TX2WOB | DWT (2015) | NP (2015) | Diam (2021) | FWT (2021) | NP (2021) | PD (2021) |
| Diam (2015) | 0.47** | -0.10* | 0.66** | 0.30** | -0.01 | 0.21** |
| DWT (2015) |  | 0.53** | 0.32** | 0.76** | 0.53** | 0.71** |
| NP (2015) |  |  | -0.23** | 0.84** | 0.93** | 0.73** |
| Diam (2021) |  |  |  | 0.27** | -0.28** | 0.03 |
| FWT (2021) |  |  |  |  | 0.85** | 0.78** |
| NP (2021) |  |  |  |  |  | 0.79** |
|  |  |  |  |  |  |  |
| TX2WSE | FWT (2021) | NP (2021) | PD (2021) |  |  |  |
| Diam (2021) | 0.27** | -0.18* | 0.19* |  |  |  |
| FWT (2021) |  | 0.74** | 0.89** |  |  |  |
| NP (2021) |  |  | 0.69** |  |  |  |
