# Supplementary material for: Pedigree-based QTL analysis of flower size traits in two multi-parental diploid rose populations
Source: Front Plant Sci. 2023 Aug 15;14:1226713. doi: 10.3389/fpls.2023.1226713 (PMC10464838; doi:10.3389/fpls.2023.1226713)
Supplement: Supplementary file 27 [file Table_9.docx]

Supplementary Table 9. List of candidate genes located in the genomic region between 60.44 to 60.95 Mbp on chromosome 1 of the Rosa chinensis genome v1.0 (Hibrand Saint-Oyant et al., 2018), as derived from the Genome Database for Rosaceae (GDR).

| ***Gene ID*** | ***Start position***  ***(bp)*** | ***End position***  ***(bp)*** | ***Gene description*** |
| --- | --- | --- | --- |
| RC1G0534700 | 60442719 | 60444870 | BCL-2-associated athanogene 1 |
| RC1G0534800 | 60451703 | 60453737 | Dihydrodipicolinate reductase |
| RC1G0534900 | 60453910 | 60455087 | - |
| RC1G0535000 | 60455089 | 60456547 | Protein of unknown function (DUF2930) |
| RC1G0535100 | 60457181 | 60458305 | F-box family protein with a domain of unknown function (DUF295) |
| RC1G0535200 | 60476458 | 60478448 | Dihydrodipicolinate reductase |
| RC1G0535300 | 60478953 | 60481333 | Protein of unknown function (DUF2930) |
| RC1G0535400 | 60509332 | 60510206 | - |
| RC1G0535500 | 60510835 | 60511396 | - |
| RC1G0535600 | 60518100 | 60519834 | - |
| RC1G0535700 | 60529050 | 60530711 | Dihydrodipicolinate reductase |
| RC1G0535800 | 60539821 | 60545790 | SEUSS-like 2 |
| RC1G0535900 | 60546240 | 60548589 | phloem protein 2-A13 |
| RC1G0536000 | 60558824 | 60562382 | S-adenosyl-L-methionine-dependent methyltransferases superfamily protein |
| RC1G0536100 | 60563180 | 60563920 | Bifunctional inhibitor/lipid-transfer protein/seed storage 2S albumin superfamily protein |
| RC1G0536200 | 60565719 | 60569533 | FAR1-related sequence 5 |
| RC1G0536300 | 60569782 | 60572716 | IQ-domain 24 |
| RC1G0536400 | 60579644 | 60580274 | Bifunctional inhibitor/lipid-transfer protein/seed storage 2S albumin superfamily protein |
| RC1G0536500 | 60580459 | 60580535 | tRNA-Val |
| RC1G0536600 | 60580555 | 60582078 | RHOMBOID-like protein 3 |
| RC1G0536700 | 60583438 | 60585319 | ribosomal protein L24 |
| RC1G0536800 | 60589533 | 60594193 | homeodomain GLABROUS 1 |
| RC1G0536900 | 60618125 | 60619805 | SEUSS-like 2 |
| RC1G0537000 | 60620581 | 60621551 | Protein of unknown function |
| RC1G0537100 | 60624672 | 60628556 | XB3 ortholog 3 in Arabidopsis thaliana |
| RC1G0537200 | 60629402 | 60630861 | centroradialis |
| RC1G0537300 | 60644934 | 60648752 | Leucine-rich repeat transmembrane protein kinase |
| RC1G0537400 | 60656752 | 60658055 | heat shock transcription factor B2A |
| RC1G0537500 | 60660279 | 60660478 | Protein of unknown function |
| RC1G0537600 | 60660480 | 60661769 | - |
| RC1G0537700 | 60664448 | 60665647 | - |
| RC1G0537800 | 60668144 | 60668755 | Sugar isomerase (SIS) family protein |
| RC1G0537900 | 60671471 | 60673830 | phosphoprotein phosphatase inhibitors |
| RC1G0538000 | 60675301 | 60678452 | GTP-binding protein 1 |
| RC1G0538100 | 60683001 | 60685107 | methyl-CPG-binding domain protein 13 |
| RC1G0538200 | 60691710 | 60692913 | - |
| RC1G0538300 | 60695871 | 60697759 | membrane-associated progesterone binding protein 3 |
| RC1G0538400 | 60698841 | 60699770 | RNI-like superfamily protein |
| RC1G0538500 | 60701339 | 60701695 | RNI-like superfamily protein |
| RC1G0538600 | 60701843 | 60702771 | - |
| RC1G0538700 | 60702853 | 60704245 | RNI-like superfamily protein |
| RC1G0538800 | 60712817 | 60716271 | NAD(P)-binding Rossmann-fold superfamily protein |
| RC1G0538900 | 60717854 | 60719882 | RNI-like superfamily protein |
| RC1G0539000 | 60720888 | 60726710 | homolog of DNA mismatch repair protein MSH3 |
| RC1G0539100 | 60729685 | 60730897 | Transducin/WD40 repeat-like superfamily protein |
| RC1G0539200 | 60735071 | 60737972 | Cleavage/polyadenylation specificity factor |
| RC1G0539300 | 60738136 | 60739260 | Tetratricopeptide repeat (TPR)-like superfamily protein |
| RC1G0539400 | 60746768 | 60748343 | myb domain protein 19 |
| RC1G0539500 | 60749136 | 60751081 | Remorin family protein |
| RC1G0539600 | 60765061 | 60765822 | Cytochrome b561/ferric reductase transmembrane protein family |
| RC1G0539700 | 60765824 | 60768381 | - |
| RC1G0539800 | 60768404 | 60769757 | Cytochrome b561/ferric reductase transmembrane protein family |
| RC1G0539900 | 60781642 | 60784122 | Cytochrome b561/ferric reductase transmembrane protein family |
| RC1G0540000 | 60794305 | 60798390 | Myosin heavy chain-related protein |
| RC1G0540100 | 60798492 | 60799716 | shortage in chiasmata 1 |
| RC1G0540200 | 60799820 | 60800690 | - |
| RC1G0540300 | 60800756 | 60804606 | shortage in chiasmata 1 |
| RC1G0540400 | 60807346 | 60810004 | CAP160 protein |
| RC1G0540500 | 60815263 | 60815877 | Protein of unknown function |
| RC1G0540600 | 60825851 | 60827398 | Pentatricopeptide repeat (PPR) superfamily protein |
| RC1G0540700 | 60829733 | 60830940 | Copper transport protein family |
| RC1G0540800 | 60832134 | 60833326 | Heavy metal transport/detoxification superfamily protein |
| RC1G0540900 | 60836051 | 60838004 | Arabidopsis thaliana protein of unknown function (DUF821) |
| RC1G0541000 | 60839173 | 60842025 | Arabidopsis thaliana protein of unknown function (DUF821) |
| RC1G0541100 | 60842243 | 60843085 | actin depolymerizing factor 7 |
| RC1G0541200 | 60843339 | 60845604 | VASCULAR-RELATED NAC-DOMAIN 6 |
| RC1G0541300 | 60846849 | 60846930 | tRNA-Arg |
| RC1G0541400 | 60853827 | 60853900 | tRNA |
| RC1G0541500 | 60854140 | 60854221 | tRNA-Arg |
| RC1G0541600 | 60854765 | 60854838 | tRNA |
| RC1G0541700 | 60855080 | 60855157 | tRNA-Arg |
| RC1G0541800 | 60858693 | 60861003 | tubulin beta 8 |
| RC1G0541900 | 60861015 | 60862590 | PAR1 protein |
| RC1G0542000 | 60870190 | 60870695 | - |
| RC1G0542100 | 60870848 | 60870954 | mir-393 |
| RC1G0542200 | 60872139 | 60877650 | RNA polymerase III subunit RPC82 family protein |
| RC1G0542300 | 60878244 | 60880279 | Protein of unknown function |
| RC1G0542400 | 60881512 | 60885039 | cytochrome P450 |
| RC1G0542500 | 60885102 | 60887069 | cytochrome P450 |
| RC1G0542600 | 60887910 | 60888628 | cytochrome P450 |
| RC1G0542700 | 60903514 | 60906299 | cytochrome P450 |
| RC1G0542800 | 60912666 | 60925379 | tripeptidyl peptidase ii |
| RC1G0542900 | 60926311 | 60931362 | Pectinacetylesterase family protein |
| RC1G0543000 | 60931730 | 60932342 | - |
| RC1G0543100 | 60935534 | 60938609 | SIGNAL PEPTIDE PEPTIDASE-LIKE 1 |
| RC1G0543200 | 60939022 | 60945391 | cleavage and polyadenylation specificity factor 100 |
| RC1G0543300 | 60946133 | 60947686 | cytochrome P450 |
